# Mitochondrial pyruvate metabolism regulates the activation of quiescent adult neural stem cells

**DOI:** 10.1126/sciadv.add5220

**Published:** 2023-03-01

**Authors:** Francesco Petrelli, Valentina Scandella, Sylvie Montessuit, Nicola Zamboni, Jean-Claude Martinou, Marlen Knobloch

**Affiliations:** ^1^ Department of Biomedical Sciences, University of Lausanne, Lausanne, Switzerland.; ^2^ Department of Cell Biology, University of Geneva, Geneva, Switzerland.; ^3^ Institute for Molecular Systems Biology, ETH Zurich, Zurich, Switzerland.

## Abstract

Cellular metabolism is important for adult neural stem/progenitor cell (NSPC) behavior. However, its role in the transition from quiescence to proliferation is not fully understood. We here show that the mitochondrial pyruvate carrier (MPC) plays a crucial and unexpected part in this process. MPC transports pyruvate into mitochondria, linking cytosolic glycolysis to mitochondrial tricarboxylic acid cycle and oxidative phosphorylation. Despite its metabolic key function, the role of MPC in NSPCs has not been addressed. We show that quiescent NSPCs have an active mitochondrial metabolism and express high levels of MPC. Pharmacological MPC inhibition increases aspartate and triggers NSPC activation. Furthermore, genetic *Mpc1* ablation in vitro and in vivo also activates NSPCs, which differentiate into mature neurons, leading to overall increased hippocampal neurogenesis in adult and aged mice. These findings highlight the importance of metabolism for NSPC regulation and identify an important pathway through which mitochondrial pyruvate import controls NSPC quiescence and activation.

## Teaser

Inhibition of pyruvate entry into mitochondria promotes the activation of adult neural stem cells and increases neurogenesis.

## INTRODUCTION

Stem cells must maintain a tight balance between quiescence, proliferation, and differentiation to sustain lifelong tissue regeneration and maintenance. This is also the case for adult neural stem/progenitor cells (NSPCs), which form newborn neurons throughout life (*1*). NSPCs are primarily quiescent in adulthood but can proliferate upon intrinsic and extrinsic stimuli (*2*). Although NSPC activation is critical for proper neurogenesis, the underlying mechanisms are still not fully understood.

Cellular metabolism has been shown to determine the activity state of stem cells (*3*, *4*), and metabolic features appear similar among different tissue-specific adult stem cells. In general, stem cells are rather glycolytic to support synthesis of cellular building blocks to sustain cell growth, while during differentiation, their metabolic profile shifts toward oxidative metabolism to generate adenosine triphosphate (*5*–*13*). Such a metabolic shift seems also important for NSPCs. Single-cell RNA sequencing (scRNA-seq) studies found decreased expression of glycolytic genes and an up-regulated expression of genes involved in oxidative phosphorylation (OXPHOS) at early stages of fate transition in adult NSPCs (*14*, *15*). These findings are supported by metabolic analyses of NSPCs under differentiation in vitro (*16*, *17*). However, recent findings suggest that when NSPCs are in a quiescent state, their metabolism might be substantially different from glycolytic, proliferating NSPCs: Quiescent NSPCs have high levels of mitochondrial fatty acid β-oxidation (FAO) and express many proteins involved in diverse aspects of mitochondrial metabolism (*18*, *19*). Furthermore, mitochondria are abundant in NSPCs, and their dynamics affects self-renewal and fate choice (*20*, *21*). Thus, mitochondrial metabolism might play a more important role for NSPC quiescence than previously anticipated.

A shift from a glycolytic to more oxidative metabolism very often requires a redirection of pyruvate, the end product of glycolysis, from lactate production toward mitochondrial oxidation in the tricarboxylic acid (TCA) cycle. The mitochondrial pyruvate carrier (MPC), a heterodimer of MPC1 and MPC2, is required for this transport (*22*–*24*). Absence of one of the two proteins leads to a loss of pyruvate transport and has a profound impact on the metabolic state of the cells (*25*, *26*). Despite its key role in linking glycolysis and mitochondrial metabolism, it remains unknown whether MPC plays a regulatory role in NSPC behavior.

We here used pharmacological MPC inhibition and genetic deletion of *Mpc1* to assess whether a disruption of pyruvate import into mitochondria would affect NSPC maintenance, activation, and differentiation. Unexpectedly, we found that quiescent NSPCs express high levels of MPC and require pyruvate import into mitochondria for the maintenance of quiescence. Inhibition of MPC triggers their activation by increasing the intracellular pool of aspartate despite a substantial decrease of TCA cycle intermediates. Furthermore, conditional MPC1-knockout (cKO)NSPCs are able to differentiate into mature neurons, indicating a high metabolic flexibility, which allows these cells to adapt their metabolism according to substrate availability. We further show that this increased activation and undisturbed differentiation of MPC1-cKO NSPCs leads to an overall increase in neurogenesis in adult and middle-aged mice.

## RESULTS

### MPC is dynamically regulated with activity state, and its transport function is required for NSPC quiescence

**Fig. 1.**
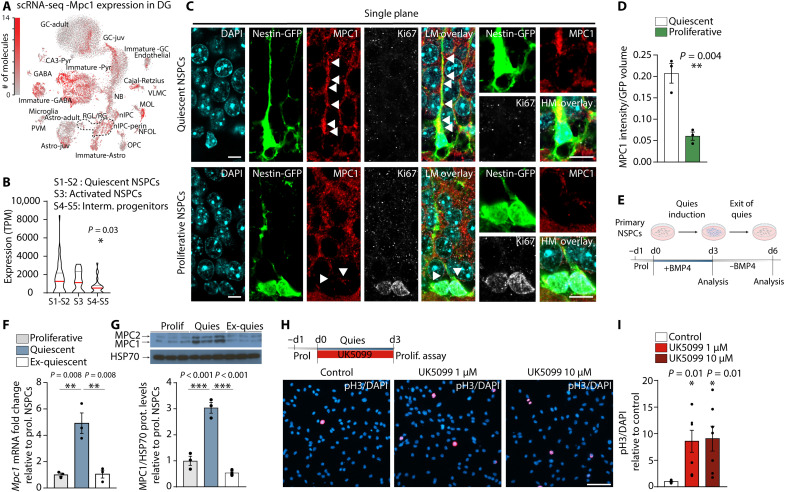
MPC is dynamically regulated with activity state, and its transport function is required for NSPC quiescence. (**A**) t-distributed stochastic neighbor embedding visualization of *Mpc1* expression in the DG of adult mice (linnarsonnlab.org/dentate). NSPCs are shown as radial glia (RG) and radial glia–like cells (RGL). (**B**) Violin plots of *Mpc1* expression [transcripts per million (TPM)] in quiescent NSPCs, activated NSPCs and intermediate progenitor cells, data queried from (*27*). Red line represents mean. *P < 0.05 [one-way analysis of variance (ANOVA) followed by post hoc test]. (**C**) Low-magnification (LM) and high-magnification confocal images of GFP (green), MPC1 (red), and Ki67 (white) immunostaining in DG of Nestin-GFP reporter mice. Scale bar, 10 μm. Arrowheads show MPC1 staining. (**D**) Quantification of MPC1 in Nestin-GFP–positive quiescent and proliferative NSPCs. Data are shown as means ± SEM. **P < 0.01, *n* = 3 mice (unpaired Student’s *t* test). (**E**) Experimental outline of quiescence induction. (**F**) Relative mRNA expression of *Mpc1* in proliferative, quiescent, and ex-quiescent NSPCs. Bars represent means ± SEM. **P < 0.01, *n* = 3 biological replicates (one-way ANOVA followed by post hoc test). (**G**) Representative Western blots of MPC1 (12 kDa), MPC2 (14 kDa), and HSP70 (70KDa) in proliferative, quiescent, and ex-quiescent NSPCs. MPC1 protein levels were normalized to mitochondrial HSP70 levels and expressed as fold change to proliferative NSPCs. Bars represent means ± SEM. ***P < 0.001, *n* = 3 biological replicates (one-way ANOVA followed by post hoc test). (**H**) Experimental outline and images of pH3 and 4′,6-diamidino-2-phenylindole (DAPI) immunostaining in quiescent NSPCs treated with UK5099. Scale bar, 50 μm. (**I**) Quantification of pH3^+^ in control or UK5099-treated quiescent NSPCs. Data represent means ± SEM. *P < 0.05. *n* = 6 coverslips from three independent experiments (one-way ANOVA followed by post hoc test).

To determine the role of MPC in NSPCs, we first analyzed the expression of *Mpc1 *in existing RNA-seq databases. We found that *Mpc1* is expressed in NSPCs and in other cell types in the dentate gyrus (DG) of adult mice (Fig. 1A) (*27*). Available images from DG sections of MPC1–green fluorescent protein (GFP) reporter mice (www.gensat.org) further showed expression of GFP in the subgranular zone of the DG (fig. S1A).

Unexpectedly, when we analyzed scRNA-seq data comprising different activity states of NSPCs in vivo (*15*), we found that *Mpc1* expression was highest in the more quiescent NSPC populations and decreased with lineage progression (Fig. 1B and fig. S1B), suggesting that the import of pyruvate into mitochondria might play a fundamental role in the regulation of NSPC quiescence. Staining for MPC1 protein in Nestin-GFP reporter mice confirmed that MPC1 is indeed highly expressed in radial glia–like quiescent NSPCs and reduced upon proliferation (Fig. 1, C and D).

To assess whether *Mpc1* expression was dependent on the activity state of NSPCs, we used cultured adult hippocampal NSPCs and applied an established in vitro system, which mimics quiescence in a reversible way (Fig. 1E) (*18*, *28*, *29*). Supporting the scRNA-seq analysis, we found that the levels of *Mpc1* mRNA and MPC1 protein were significantly higher in quiescent NSPCs compared to active/proliferating NSPCs (Fig. 1, F and G). *Mpc2*/MPC2 followed the same pattern (Fig. 1G and fig. S1C). When the artificial quiescence state of NSPCs was reverted back to a proliferative state, *Mpc1*/MPC1 and MPC2 levels were significantly reduced to the levels of proliferative NSPCs (Fig. 1, F and G, and fig. S1C), indicating that MPC levels are dynamically regulated with activity state. OXPHOS complexes followed the same pattern, with increased protein levels in quiescent NSPCs (fig. S1, D and E). Furthermore, morphometric analysis of mitochondria revealed that quiescent NSPCs contained primarily fused and elongated mitochondria, which are indicative of highly active OXPHOS (*30*, *31*), whereas proliferative NSPCs had more fragmented mitochondria (fig. S1, F and G). Together, these findings show that quiescent NSPCs have high levels of MPC and an elongated mitochondrial network.

Given the high MPC levels, we next tested whether inhibiting MPC would affect the quiescence of these cells. We used the specific MPC inhibitor UK5099 (*32*) either during quiescence induction (Fig. 1, H and I) or after established quiescence (fig. S1, H and I). Notably, UK5099 significantly increased the number of cycling and dividing Ki67 and phospho-histone 3 (pH3)–positive NSPCs, suggesting that MPC activity is indeed required for maintenance of quiescence and that its blockage leads to NSPC activation in vitro. To confirm these findings, we isolated and expanded NSPCs from adult *Mpc1* floxed mice [*Mpc1* fl/fl (*33*)] and wild-type (WT) control mice. Infection with a Cre-GFP retrovirus (RV) during proliferation led to a clear reduction in MPC1 protein in the *Mpc1* fl/fl NSPCs (hereafter called MPC1 cKO) compared to virus-treated *Mpc1* WT NSPCs (MPC1 WT), as shown by immunohistochemistry (fig. S1J). Ablation of *Mpc1* significantly increased the number of proliferating NSPCs under quiescence conditions, confirming the findings obtained with UK5099 (fig. S1, K and L).

### *Mpc1* deletion in NSPCs in vivo leads to increased numbers of progeny and triggers NSPC proliferation

**Fig. 2.**
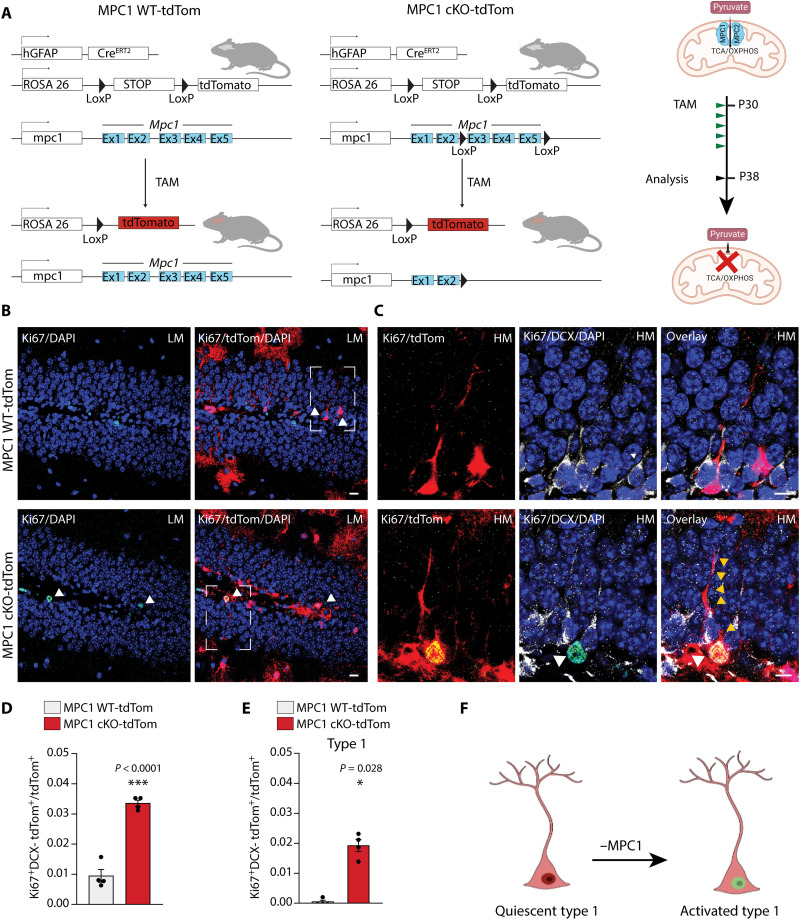
*Mpc1* deletion in NSPCs in vivo leads to increased numbers of progeny and triggers NSPC proliferation. (**A**) Generation of MPC1 cKO-tdTom and MPC1 WT-tdTom mice and experimental timeline of tamoxifen (TAM) injections (green arrows) and data analysis (black arrow). (**B**) LM confocal images of TAM-induced td-Tom recombination (red), Ki67 (green), and DAPI (blue) immunostaining at P38 in the DG. Scale bars, 10 μm. (**C**) High-magnification (HM) confocal images of TAM-induced td-Tom recombination (red), Ki67 (green), doublecortin (DCX; white), and DAPI (blue) immunostaining at P38 in the DG. White arrowheads show the colocalization of Ki67 and tdTom in a single confocal plane. Yellow arrowheads show type 1 cell processes. Scale bars, 10 μm. (**D**) Quantification of Ki67-positive (Ki67^+^) tdTom^+^ cells over total number of tdTom^+^ cells in the DG. Data are shown as means ± SEM. ***P < 0.001, *n* = 4 mice per group (unpaired Student’s *t* test). (**E**) Quantification of type 1 Ki67*^+^* DCX- tdTom^+^ cells over the total number of tdTom^+^ cells. Data are shown as means ± SEM. *P < 0.05, *n* = 4 mice per group (unpaired Mann-Whitney *t* test). (**F**) Illustration shows type 1 cell activation following *Mpc1* deletion.

We next addressed whether MPC is important for the maintenance of quiescent NSPCs in vivo. To delete *Mpc1* in adult NSPCs, we crossed *Mpc1* fl/fl mice with a tamoxifen-inducible glial fibrillary acidic protein (GFAP)–Cre recombinase line [hGFAP-CreERT2 (*34*)]. To visualize the recombined cells and their progeny, we additionally crossed these inducible *Mpc1* cKO mice with an inducible tdTomato lineage tracing line [tdTom (*35*)], resulting in triple transgenic mice hereafter called MPC1 cKO-tdTom mice (Fig. 2A). Control mice were generated the same way but carried WT alleles of the *Mpc1* gene (MPC1 WT-tdTom mice). Cre expression was induced by administration of tamoxifen at postnatal day 30 (P30), leading to the deletion of *Mpc1* in GFAP-expressing cells from cKO-tdTom mice, including NSPCs (Fig. 2A).

To determine the effect of *Mpc1* deletion on the activation of NSPCs in vivo, we analyzed the number of tdTomato-positive cells at P38 (Fig. 2A), a time point where MPC1 protein was clearly reduced in MPC1 cKO-tdTom NSPCs (fig. S2B). While the total number of tdTom-positive (tdTom^+^) cells was not increased at P38, i.e., 8 days after the first tamoxifen injections (fig. S2C), the proportion of activated/proliferative tdTom^+^ cells was significantly increased, as revealed by the cell cycle marker protein Ki67 (Fig. 2, B to D). As several subtypes of NSPCs in the DG can proliferate, we categorized the Ki67^+^/tdTom^+^ cells according to the previously described nomenclature (*36*) into type 1 cells (radial glia–like cells) and type 2a cells (no radial process, more proliferative progenitors). Type 2a cells were further distinguished from committed neuroblasts by the absence of the protein doublecortin (DCX), an immature neuronal marker (fig. S2A). Whereas the number of Ki67^+^ tdTom^+^ type 2a cells was comparable between control and MPC1 cKO-tdTom mice (fig. S2D), the number of Ki67^+^tdTom^+^ cell type 1 cells was significantly increased in the KO mice (Fig. 2, C and E), indicating a substantial activation of quiescent NSPCs upon *Mpc1* deletion (Fig. 1F). 

Together, these data show that *Mpc1* deletion in NSPCs leads to an increased activation and proliferation of NSPCs, resulting in higher numbers of progeny generated. These findings are in line with the increased activation of quiescent NSPCs seen in vitro upon MPC blockage and *Mpc1* cKO (Fig. 1, H and I, and fig. S1, K and L).

### Increased lactate levels are not essential for the activation of quiescent NSPCs

**Fig. 3.**
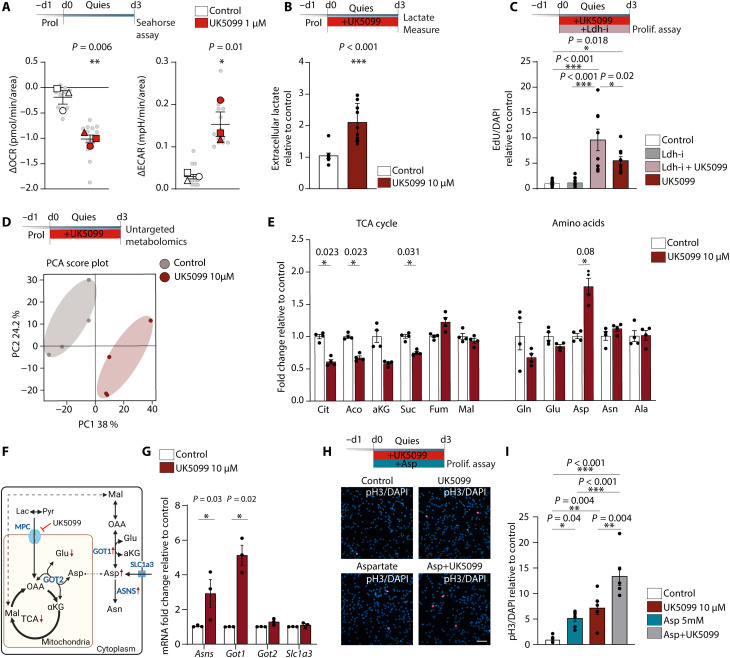
MPC inhibition in quiescent NSPCs leads to increased intracellular aspartate levels. (**A**) Experimental outline. Bottom: OCR and ECAR changes after acute MPC inhibition with 1 μM UK5099. *P < 0.05, **P < 0.01, *n* = 3 biological replicates; gray dots represent individual Seahorse wells (unpaired Student’s *t* test). (**B**) Extracellular lactate in quiescent NSPCs. Data represent means ± SEM, ***P < 0.001, *n* = 9 samples from three independent experiments (unpaired Student’s *t* test). (**C**) Top: Experimental outline. Bottom: Quantification of 5-ethynyl-2'-deoxyuridine-positive cells in control-, Ldh-i–, Ldh-i + UK5099–, and UK5099-treated quiescent NSPCs. Data represent means ± SEM *P < 0.05, ***P < 0.001. *n* = 9 coverslips from three independent experiments (**D**) Top: Experimental outline. Bottom: Principal components analysis (PCA) of untargeted metabolomics from control- and UK5099-treated quiescent NSPCs. Gray, control-treated cells; red, UK5099-treated cells. *n* = 4 biological replicates. (**E**) Relative intensity of selected metabolites in control- and UK5099-treated quiescent NSPCs. Bars represent means ± SEM normalized to control condition. *P < 0.05*, n* = 4 biological replicates. Cit, citrate; Aco, aconitate; aKG, alpha-ketoglutarate; Suc, succinate; Fum, fumarate; Mal, malate; Gln, glutamine; Glu, glutamate; Asp, aspartate; Asn, asparagine; and Ala, alanine. (**F**) Main metabolic pathways involved in the synthesis and utilization of aspartate. OAA, oxaloacetate. (**G**) Relative mRNA expression of *Asns*, *Got1*, *Got2*, and *Slc1a3* in control- and UK5099-treated NSPCs; bars represent means ± SEM. *P < 0.05, *N* = 3 independent experiments. Statistics were computed on ΔCT values (unpaired Student’s *t* test). (**H**) Top: Experimental outline. Bottom: Images of pH3 and DAPI immunostaining in quiescent NSPCs treated with UK5099, aspartate and UK5099 + aspartate. Scale bar, 50 μm. (**I**) Quantification of pH3-positive cells in control-, aspartate-, UK5099- and aspartate + UK5099–treated quiescent NSPCs. Data represent means ± SEM. *P < 0.05, **P < 0.01, ***P < 0.005. *n* = 6 coverslips from two independent experiments (one-way ANOVA followed by post hoc test).

We next assessed the metabolic profile of quiescent NSPCs by using Seahorse technology. A fuel flexibility test showed that the oxygen consumption rate (OCR) in quiescent NSPCs depends mainly on glucose. However, glutamine and fatty acids also contributed as oxidative fuel sources (fig. S3A), suggesting that quiescent NSPCs are flexible in their use of substrates for energy and biosynthetic metabolism. OCR was markedly impaired after acute injection of UK5099 under basal conditions in quiescent NSPCs (Fig. 3A), while the inhibition of FAO and glutaminolysis using etomoxir and bis-2-(5-phenylacetamido-1,3,4-thiadiazol-2-yl)ethyl sulfide (BPTES), respectively, decreased OCR to a lesser extent (fig. S3B). These data suggest that quiescent NSPCs use pyruvate as the main substrate for mitochondrial respiration and that at least a part of the pyruvate produced by glycolysis is transported into mitochondria.

Acute inhibition with UK5099 also resulted in a significant increase in the extracellular acidification rate (ECAR; Fig. 3A), whereas blocking FAO and glutaminolysis had no effect on ECAR (fig. S3C). These results suggest increased lactate secretion upon inhibition of MPC, which we confirm by direct measurement of lactate in the medium of quiescent NSPCs treated with UK5099 (Fig. 3B). Previous studies have shown that exogenous lactate can promote NSPC proliferation (*37*–*39*). We therefore speculated that higher extracellular lactate levels might be the cause of increased proliferation after MPC inhibition. We therefore tested whether addition of lactate during quiescence induction could mimic the effect of UK5099. However, the proliferation rate of quiescent NSPCs did not change with the addition of lactate (fig. S3D), although the same dose of lactate was sufficient to increase the proliferation of NSPCs under proliferating conditions (fig. S3E). To demonstrate that an increased lactate concentration per se is not sufficient to explain the effects of UK5099 on quiescent NSPCs, we next used an established lactate dehydrogenase inhibitor [GSK 2837808A (*40*), here called Ldh-i], which prevents the conversion of pyruvate to lactate. The lactate concentration in the medium of quiescent NSPCs treated with both Ldh-i and UK5099 was significantly lower than in cells treated with UK5099 alone (fig. S3F), indicating that Ldh-i reduced lactate production as intended. If the conversion of pyruvate to lactate were required for the increased proliferation after MPC inhibition, then concomitant treatment with Ldh-i should at least partially prevent the UK5099 effect. However, we found no difference in the proliferation of NSPCs under quiescence condition when treated with UK5099 alone or with UK5099 and Ldh-i simultaneously (Fig. 3C). Together, these data suggest that elevated lactate levels are not essential for the activation of quiescent NSPCs.

### MPC inhibition in quiescent NSPCs leads to increased intracellular aspartate levels

To analyze the metabolic changes resulting from inhibition of mitochondrial pyruvate import in quiescent NSPCs, we performed untargeted metabolomics on NSPCs cultured in the presence or absence of UK5099 during the induction of quiescence (Fig. 3D). Principal components analyses revealed that samples clustered according to the treatment, suggesting that MPC inhibition drives profound metabolic changes in quiescent NSPCs (Fig. 3D and fig. S3G). Pathway enrichment analysis showed that UK5099 significantly affected TCA cycle and amino acid metabolism (fig. S3H). While intermediates of glycolysis were not significantly changed (fig. S3I), TCA intermediates, in particular, citrate and succinate levels, were clearly reduced compared to untreated cells (Fig. 3E). Notably, malate and fumarate were not significantly different. Furthermore, MPC inhibition led to a marked increase in intracellular aspartate (Fig. 3E). Overall, these measurements indicate an impairment in TCA activity and a rewiring of amino acid metabolism leading to high aspartate levels.

The intracellular pool of aspartate is maintained by the activity of two transaminases, the cytosolic enzyme glutamic-oxaloacetic transaminase 1 (GOT1) and the mitochondrial GOT2. GOT1 uses aspartate to generate glutamate and oxaloacetate, while GOT2 uses these two substates to synthesize α-ketoglutarate and aspartate (Fig. 3F). We thus measured mRNA expression of the two transaminases. *Got1* was significantly increased in quiescent NSPCs treated with UK5099 (Fig. 3G), whereas *Got2* was only slightly increased. In addition, asparagine synthase (*Asns*), which converts aspartate to asparagine, was also increased (Fig. 3G). These data suggest that increased expression of *Got* transaminases upon MPC inhibition contributes to the increased aspartate levels.

Cells can also uptake aspartate through the glutamate-aspartate plasma membrane transporter solute carrier family 1 member 3 (SLC1A3, also known as GLAST) (Fig. 3F). Quiescent NSPCs express already high levels of this transporter (*41*), and no further increase occurred upon UK5099 treatment (Fig. 3G). Exogenous aspartate or overexpression of SLC1A3 has been shown to be sufficient to support proliferation of cells lacking electron transport chain (ETC) activity (*42*, *43*). To test whether the measured increase in aspartate upon MPC inhibition could be responsible for the activation of quiescent NSPCs, we treated NSPCs with different concentrations of aspartate during quiescence induction and observed a significant increase in the number of proliferating, pH3-positive cells (Fig. 3, H and I, and fig. S3J). Moreover, simultaneous treatment with UK5099 and 5 mM aspartate further increased the ratio of pH3-positive NSPCs (Fig. 3, H and I). Beyond its role as an amino acid in proteins, aspartate is also required for nucleotide synthesis (*44*). We found that some of the nucleotide synthesis enzymes are indeed up-regulated with UK5099 treatment (fig. S3K), indicating that increased aspartate levels might be used to promote nucleotide synthesis. Together, these data show that inhibition of MPC causes an increase in aspartate levels in quiescent NSPCs, which could result from increased GOT activity and/or increased import. This increase in aspartate is sufficient to drive NSPC activation.

### MPC-deficient NSPCs can generate neurons through a shift in their metabolism

**Fig. 4.**
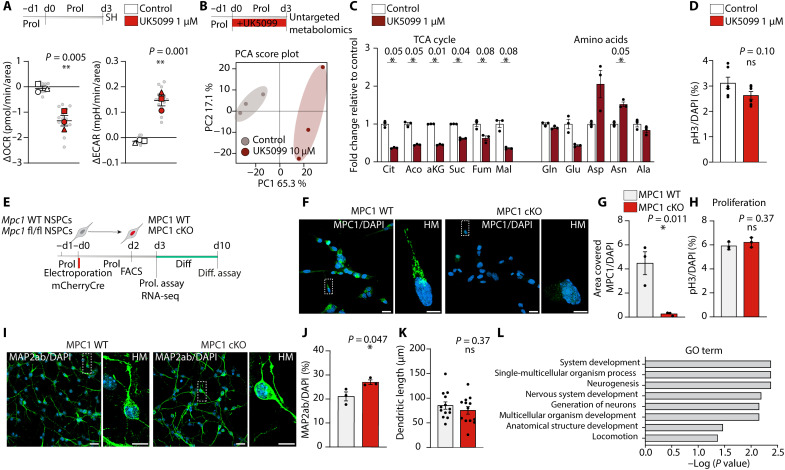
MPC-deficient NSPCs can generate neurons through a shift in their metabolism. (**A**) Experimental outline. Bottom: OCR and ECAR measurements in control and 1 μM UK5099-treated NSPCs. Bars represent means ± SEM. **P < 0.01. *n* = 3 biological replicates (unpaired Student’s *t* test), gray dots represent individual wells. (**B**) Top: Experimental outlines. Bottom: PCA of untargeted metabolomics from control- and UK5099-treated proliferative NSPCs. *n* = 4 biological replicates. (**C**) Relative intensity of selected metabolites in control- and UK5099-treated cells. Bars represent means ± SEM. * adjusted *P* value <0. 1, *n* = 3 biological replicates. (**D**) Quantification of pH3^+^ cells in control- and UK5099-treated proliferative NSPCs. Data represent means ± SEM. Not significant (ns), *n* = 6 coverslips from two independent experiments. (unpaired Student’s *t* test). (**E**) Illustration shows the *MPC1* cKO system in cultured NSPCs. (**F**) LM and HM confocal images of MPC1 (green) and DAPI (blue) immunostaining in MPC1 WT and MPC1 cKO NSPCs. Scale bar, LM, 20 μm; HM, 5 μm. (**G**) Quantification of MPC1 intensity in MPC1 WT and MPC1 cKO NSPCs. *P < 0.05 *n* = 3 electroporations, data show means ± SEM, unpaired Student’s *t* test. (**H**) Quantification of pH3^+^ cells in MPC1 WT and MPC1 cKO NSPCs. Data represent means ± SEM. *n* = 3 electroporations. (**I**) LM and HM confocal images of neurons (MAP2ab, green) and DAPI (blue). Scale bar, LM, 20 μm; HM, 5 μm. (**J**) Quantification of MAP2ab-positive cells in MPC1 WT and MPC1 cKO cells. *P < 0.05, *n* = 3 electroporations, means ± SEM (unpaired Student’s *t* test). (**K**) Quantification of dendritic length of MAP2ab-positive neurons. *n* = 13 neurons per group (unpaired Student’s *t* test). (**L**) GO term analysis of the up-regulated genes in MPC1-cKO NSPCs.

Once activated, NSPCs can proliferate and eventually differentiate into neurons. We therefore next tested the effect of UK5099 in already active/proliferative NSPCs. Similar to quiescent NSPCs, active NSPCs showed decreased OCR and increased ECAR upon treatment with UK5099 (Fig. 4A), suggesting that these cells also use pyruvate to drive OXPHOS despite lower levels of MPC1 and MPC2 than quiescent NSPCs (Fig. 1, F and G). Untargeted metabolomics showed a metabolic shift (Fig. 4B), with a significant decrease of TCA intermediates (Fig. 4C) and a shift toward increased production of aspartate and asparagine with UK5099 treatment (Fig. 4C), while no changes in the intermediates of glycolysis were found (fig. S4A). However, despite these changes in mitochondrial metabolism, UK5099 did not alter the proliferation rate of already active NSPCs (Fig. 4D).

Neuronal differentiation is associated with an increased expression of genes involved in OXPHOS (*14*, *15*). As glucose- or lactate-derived pyruvate is thought to be the major fuel for OXPHOS during differentiation, MPC-mediated pyruvate import should thus also play an important role during NSPC differentiation. We therefore tested whether active NSPCs could differentiate into neurons in the presence of UK5099. We found a clear reduction in the total number of cells after 7 days of differentiation in the presence of UK5099 (fig. S4B), indicating that mitochondrial pyruvate might regulate cell survival or cell cycle exit during the differentiation process. However, unexpectedly, UK5099-treated NSPCs were still able to give rise to microtubule-associated protein 2ab (MAP2AB)–positive neurons in a similar ratio to total cell numbers as untreated NSPCs (fig. S4, C and D). These data highlight a large metabolic flexibility of NSPCs, which seem to use other metabolic pathways, in particular, amino acid metabolism, to sustain their energy needs for proliferation and differentiation when pyruvate import into mitochondria is disrupted.

To confirm these important findings, we used NSPCs from *Mpc1* fl/fl mice and WT control mice and induced Cre-mediated recombination through electroporation of an mCherry-Cre–expressing plasmid (Fig. 4E) and obtained purely transfected cell populations by fluorescence-activated cell sorting (FACS; fig. S4E). Three days after Cre-mediated deletion of Mpc1, MPC1 protein levels were strongly reduced in proliferating MPC1 cKO NSPCs compared to MPC1 WT NSPCs (Fig. 4, F and G). Similar to UK5099-treated active NSPCs, *Mpc1* deletion did not affect proliferation of activate NSPCs (Fig. 4H). We next assessed whether MPC1 cKO NSPCs were able to differentiate into neurons. While we also found a similar reduction in total cell numbers as in UK5099-treated NSPCs after 7 days of differentiation (fig. S4F), the deletion of *Mpc1* in active NSPCs did not affect the differentiation and the maturation into MAP2AB-positive neurons. MPC1-cKO NSPCs generated even slightly more MAP2AB-positive neurons per total number of cells compared to control NSPCs (Fig. 4, I to K).

To get further insights into the increased neuron production, we next performed RNA-seq on active MPC1 WT and MPC1 cKO NSPCs. Commonly used NSPC markers were comparable between the two groups (fig. S4, G to I), indicating that MPC1 cKO cells retained the main features of NSPCs despite the lack of MPC1. Among the differentially expressed genes, 160 genes were up-regulated, and 34, including *Mpc1*, were down-regulated in the MPC1 cKO NSPCs (fig. S4J). Gene ontology (GO) analysis of the up-regulated genes showed a clear enrichment of GO terms involved in neuronal differentiation (Fig. 4L), while the down-regulated genes did not enrich in a specific GO category. Together, these results indicate that blocking the import of pyruvate into the mitochondria for OXPHOS does not affect neuronal differentiation of NSPCs, contrary to what would have been predicted.

### *Mpc1* deletion in NSPCs increases neurogenesis in vivo

**Fig. 5.**
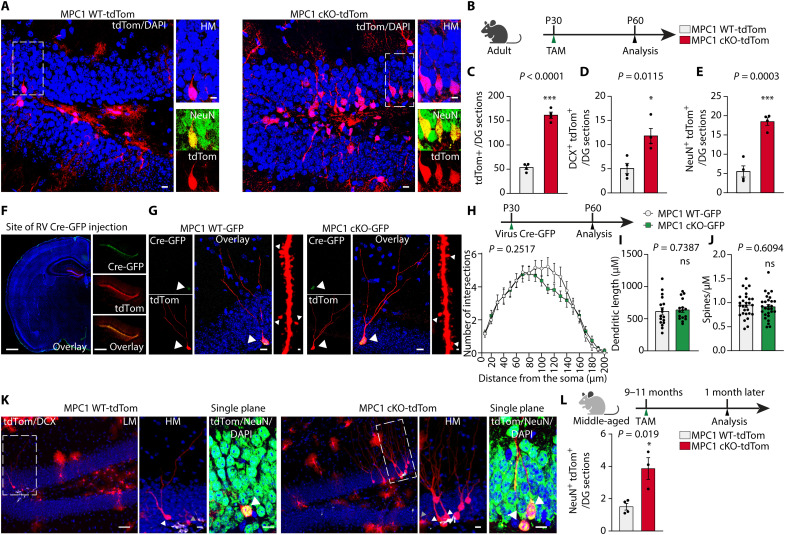
*Mpc1* deletion in NSPCs increases neurogenesis in vivo. (**A**) LM and HM confocal images of TAM-induced tdTom recombination (tdTom, red), NeuN (green), and DAPI (blue) immunostaining. Scale bars, 10 μm. (**B**) Experimental scheme. (**C**) Quantification of tdTom^+^ cells per DG sections. Data are shown as means ± SEM. ***P < 0.001, *n* = 4 mice per group (unpaired Student’s *t* test). (**D**) Quantification of DCX + tdTom^+^ cells per DG sections. Data show means ± SEM. *P < 0.05, *n* = 4 mice per group (unpaired Student’s *t* test). (**E**) Quantification of NeuN+tdTom^+^ cells. Data show means ± SEM. ***P < 0.001, *n* = 4 mice per group (unpaired Student’s *t* test). (**F**) Images of in vivo injections of RV Cre-GFP in the DG. Scale bars, 250 μm. (**G**) Confocal images of Cre-induced td-Tom + newborn neurons. Arrowheads indicate the colocalization between nuclear GFP and tdTom signal. Scale bars, 10 μm. Confocal image of spines in newborn neurons. Scale bars, 1 μm. (**H**) Top: Experimental scheme. Bottom: Sholl analysis of dendritic complexity of newborn neurons. *n* = 4 mice per group. (**I**) Quantification of the dendritic length of newborn neurons. *n* = 4 mice per group (unpaired Student’s *t* test). (**J**) Spine density of newborn neurons. *n* = 4 mice per group. (**K**) LM and HM confocal images and a single plane image of TAM-induced td-Tom recombination (red), DCX (white), NeuN (green), and DAPI (blue) immunostaining. Scale bars, 10 μm. (**L**) Top: Experimental scheme. TAM injection (green arrow) at 9 to 11 months (“middle-aged mice”), and data analysis after 1 month (black arrow). Bottom: Quantification of NeuN+dTom^+^ cells per DG sections. Data show means ± SEM. *P < 0.05, *n* = 3 to 4 mice per group (unpaired Student’s *t* test).

We next tested whether deletion of *Mpc1* in adult NSPCs in vivo would affect their differentiation capacity 30 days after the first tamoxifen injection (Fig. 5B). Cre-mediated *Mpc1* deletion at P30 led to a significant reduction in *Mpc1* mRNA and MPC1 protein in the hippocampus, as shown by quantitative reverse transcription polymerase chain reaction (qRT-PCR) and Western blot on whole hippocampal tissue of MPC1 cKO-tdTom and control mice at P60 (fig. S5, A to C). MPC2 was also down-regulated (fig. S5C), as has been previously shown in other MPC1 KO cell types (*11*, *45*–*48*).

Immunohistological analyses showed that the overall numbers of tdTom^+^ cells in the DG were significantly increased in MPC1 cKO-tdTom mice compared to control mice 1 month after *Mpc1* deletion (Fig. 5, A and C). This increase was due to increased numbers of neuroblasts and immature neurons marked by DCX^+^/tdTom^+^ (Fig. 5D and fig. S5D), as well as increased numbers of mature newborn neurons (NeuN^+^/tdTom^+^) (Fig. 5, A and E). These data show that the generation of newborn neurons in vivo is not impaired by *Mpc1* deletion in NSPCs. On the contrary, the lack of MPC1 in NSPCs even led to a significant increase in the generation of newborn neurons.

To address whether the increased neurogenesis was due to a cell-autonomous *Mpc1* cKO effect in already activated NSPCs, we performed stereotaxic injection of an RV encoding a Cre-recombinase fused to a GFP fluorophore into the DG of mice carrying floxed or WT *Mpc1* alleles crossed to the tdTom lineage tracing line (Fig. 5F). As previously reported, this viral Cre-delivery strategy targets only activated NSPCs (*49*). This approach results in MPC1-deficient NSPCs (MPC1 cKO-GFP) or control NSPCs (MPC1 WT-GFP) in an otherwise unaltered microenvironment, and all progeny are labeled with tdTom and GFP. One month after virus injection, mature neurons were found in both MPC1 cKO-GFP and MPC1 WT-GFP mice (Fig. 5G). Despite the lack of MPC1, the neurons in the MPC1 cKO-GFP mice had similar morphology to the neurons in MPC1 WT-GFP mice, as assessed by Scholl analysis and dendritic length measurements (Fig. 5, H and I). Similarly, total spine numbers were not changed (Fig. 5J), and the maturity of the spines was normal (fig. S5E). These astonishing findings show that even with disrupted oxidation of glucose-derived pyruvate, NSPCs can give rise to normal mature newborn neurons. This suggests that NSPCs and their progeny display metabolic flexibility and can compensate for impaired pyruvate import.

### *Mpc1* deletion also increases neurogenesis in advanced age mice

Hippocampal neurogenesis drastically drops with age (*50*), with more than fourfold reduction of cycling NSPCs at 9 months compared to 2 months of age (*51*). Several recent studies suggest that this is due to increased dormancy of NSPCs (*52*–*54*). When querying scRNA-seq data from a recent publication that compared dormant, resting, and proliferating NSPCs in vivo (*55*), we found that *Mpc1* and *Mpc2* were most highly expressed in dormant cells and lowest in proliferating NSPCs (fig. S5F). As Mpc1 deletion leads to an activation of quiescent NSPCs in young adult mice (Fig. 2, C, E, and F), we next tested whether deletion of *Mpc1* would have similar effects on dormant NSPCs from older animals. *Mpc1* deletion was induced in 9- to 11-month-old MPC1 cKO-tdTom mice, and the number of newborn neurons was assessed 30 days later. The number of tdTom^+^ neurons was significantly increased in the aged MPC1 cKO tdTom mice compared to the MPC1 WT tdTom mice (Fig. 5, K and L), suggesting that in mice with advanced aged, NSPCs are also activated upon *Mpc1* ablation, leading to an increase in neurogenesis.

## DISCUSSION

**Fig. 6.**
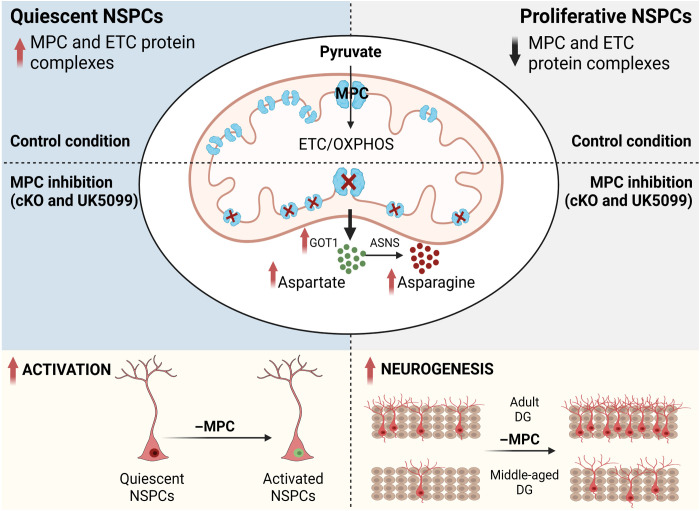
Graphical summary of the presented data.

One of the most important regulatory steps of adult neurogenesis is the decision of NSPCs to maintain quiescence or enter an active state. This decision results from intrinsic and extrinsic instructions, which are still not fully understood (*2*). We here show that the maintenance of quiescence in adult NSPCs is dependent on mitochondrial pyruvate metabolism and requires MPC activity. MPC, which imports pyruvate into the mitochondria, is highly expressed in adult NSPCs, and its loss of function leads to an activation of quiescent NSPCs and increased neurogenesis (summarized in Fig. 6). Recent studies have shown increased proliferation upon *Mpc1* deletion in other stem cells, such as intestinal and hair follicle stem cells (*10*, *11*). Furthermore, tumor cells slowed down proliferation after overexpression of *Mpc1* (*48*, *56*), suggesting that there might be a common regulation of cellular activity by MPC.

Given the general idea that stem cells are mostly glycolytic, whereas their differentiated progeny use OXPHOS, the involvement of MPC in the quiescence of NSPCs shown here is unexpected as this carrier is generally considered a marker of oxidative metabolism. Our results suggest that quiescent type 1 cells display a more oxidative metabolism than commonly thought, and the import of pyruvate into the mitochondria is necessary to maintain their quiescent state. Accordingly, we and others have reported that these cells have an elaborate mitochondrial network and can use fatty acids to fuel OXPHOS (*14*, *18*–*21*, *57*). A possible explanation for these discrepancies comes from a recent study comparing proteomic and transcriptomic levels of several metabolic proteins: Wani and colleagues (*19*) could demonstrate that changes in TCA, FAO, and OXPHOS proteins were poorly reflected at the transcriptional levels, while glycolytic enzymes showed better correlation between protein and mRNA expression. Our findings on the importance of MPC for quiescent NSPCs opens different perspectives to better understand the maintenance of quiescence from a metabolic point of view.

The detailed molecular mechanisms by which mitochondrial pyruvate controls type 1 stem cell proliferation remain to be elucidated. Although lactate levels clearly change upon MPC inhibition, our experiments using lactate supplementation and inhibition of lactate dehydrogenase suggest that increased concentration of lactate are not essential for the activation of NSPCs. Lactate production triggers the activation of hair follicle stem cells (*10*), indicating that the effect driven by lactate on the activation and proliferation depends on the stem cell types. The inhibition of pyruvate transport into mitochondria has been shown to induce a metabolic shift toward oxidation of other substrates, in particular, amino acids, which in turn rewire the metabolism of the cells (*46*, *58*, *59*). We also observe major metabolic changes when blocking mitochondrial pyruvate import, which led to the activation of quiescent NSPCs. We show that inhibition of MPC induces an increase of intracellular aspartate despite a significant decrease in OCR and TCA metabolites. Aspartate can have different sources, thus a metabolic flux analysis would be required to elucidate the detailed mechanism by which NSPCs increase their intracellular aspartate levels when treated with UK5099. Using metabolic flux analyses, other studies have shown that MPC inhibition in different cell types leads to an increase of aspartate synthesis through mitochondrial oxidation of glutamine or glutamate (*46*, *58*, *59*). Whether this is also the case in NSPCs remains to be determined. Intriguingly, we also found that MPC inhibition induces a significant increase of Got1 expression and a slight increase of Got2, two key components of the malate-aspartate shuttle system. These data suggest that quiescent NSPCs might up-regulate the malate-aspartate shuttle upon MPC inhibition to transfer cytosolic NADH [reduced form of nicotinamide adenine dinucleotide (NAD^+^)] into mitochondria for energy production and to regenerate NAD^+^ to balance the cellular NAD^+^/NADH pool. Since it has been shown that in the context of a decline in ETC activity, the recycling of NAD^+^ is necessary for cell proliferation (*60*), the malate aspartate shuttle activation might also be important in cells lacking MPC function. ETC deficiency can also lead to a reversal of GOT1 activity, leading to GOT1-dependent reductive aspartate synthesis, as, for instance, shown by two recent studies (*42*, *43*). In quiescent NSPC lacking MPC activity, the ETC is expected to be functional. However, we cannot exclude that GOT1 activity could also be reversed because of fewer electrons feeding complex I of ETC and decreased OXPHOS.

Nevertheless, aspartate per se is a key amino acid for cell proliferation not only for protein synthesis but also for purine nucleotide and pyrimidine nucleotide synthesis (*43*). Exogenous aspartate is sufficient to sustain proliferation even in the absence of a functioning ETC (*43*) and is also a limiting metabolite for cancer cell proliferation (*61*). Furthermore, aspartate levels have been shown to directly influence hematopoietic stem cell proliferation (*44*). Our findings that the addition of exogenous aspartate mimics the effect of UK5099 on quiescence exit of NSPCs are thus in line with these previous findings. The additive effect of aspartate and UK5099 suggest that both mechanisms, aspartate uptake and/or aspartate biosynthesis, contribute to the intracellular pool of aspartate. Despite its clear influence on NSPC activation, it remains to be clarified how exactly increased aspartate is exerting its effect. A possible explanation is that aspartate is used for the synthesis of purine/pyrimidine and asparagine, similar to findings in hematopoietic stem cells (*44*). Our metabolomic and mRNA expression data point in this direction, showing an increase of asparagine synthetase and asparagine synthesis as well as slight increase in nucleotide synthesis enzymes in quiescent NSPCs upon MPC inhibition. The additive effect of aspartate and UK5099 could also indicate that besides increasing intracellular aspartate, MPC inhibition might have additional effects on the activation of quiescent NSPCs.

A shift in the balance of NSPC quiescence and activation is expected to affect net neurogenesis as the production of new neurons is the default fate of activated adult hippocampal NSPCs (*1*). Accordingly, we found a substantial increase in the number of newborn neurons upon *Mpc1* deletion. However, given the commonly accepted view that most neurons heavily rely on glucose- and lactate-derived pyruvate for OXPHOS (*62*, *63*), such an increase in morphologically normal neurons is astonishing, as one would expect that newborn neurons cannot mature properly without pyruvate oxidation. These results suggest that newborn neurons might have a higher metabolic flexibility than previously thought if their preferential metabolic pathway is blocked. In line with this, it has been shown that several types of mature neurons are indeed able to sustain their functions by shifting their metabolism to other substrates, which can fuel OXPHOS when MPC is inhibited (*59*, *64*, *65*). We show that MPC1 cKO NSPCs are able to generate mature neurons in vitro and in vivo. These findings highlight a new form of metabolic plasticity used by NSPCs to differentiate into mature neurons. MPC1 cKO further seems to prime NSPCs toward neuronal differentiation, as our RNA-seq data suggest. These data illustrate that the metabolic state might directly influence fate decision. Whether this neuronal priming is achieved via epigenetic modifications in MPC1 cKO NSPCs remains to be determined.

Hippocampal neurogenesis decreases strongly with age because of terminal differentiation and increased deep quiescence of NSPCs (*52*, *53*). Our data show that MPC deletion is also sufficient to wake up these more dormant NSPCs in aged mice, resulting in increased number of newborn neurons. Supporting these findings, we found that *Mpc1* is highest in dormant NSPCs in a recent scRNA-seq study comparing different states (*55*), which is also in line with our in vitro quiescence data.

In conclusion, we have shown that quiescence of NSPCs is an actively maintained state, requiring MPC-mediated import of pyruvate into the mitochondria. MPC inactivation leads to activation of quiescent NSPCs and subsequently increased neurogenesis, even in aged mice. Our findings thus describe a mechanism through which mitochondrial metabolism controls NSPC function.

## MATERIALS AND METHODS

### Animals

All studies were approved by the Service de la consommation et des affaires vétérinaires of Geneva in Switzerland. Mice were group-housed with littermates in standard housing on a 12:12-hour light/dark cycle. Nestin-GFP reporter mice (*66*) have been obtained from S. Jessberger (University of Zürich), hGFAP-CreERT2 mice (*34*) from N. Toni (University Hospital Lausanne, CHUV), *Mpc1* fl/fl mice (*33*) from E. Taylor (University of Iowa), and tdTom fl-STOP-fl [Ai14 (*35*)] from I. Rodriguey (University of Geneva). All mice were on a C57BL/6 background. Genotyping was performed on DNA extracted from phalange biopsies using the following primers: hGFAP-CreERT2: F-CAG GTT GGA GAG GAG ACG CAT CA, R-CGT TGC ATC GAC CGG TAA TGC AGG C; MPC1 fl/fl: F1-CCT ATT CTC TAG AAA GTA TAG GAA CTT CGT CGA, F2-GTG AGC CCA GAG CTA CGA AGG ATC GGC, F3-GGA AAG AAA AAG GTG TCC AAT TTT AGC TCT GCA; tdTom fl STOP-fl: F 5′-CTG TTC CTG TAC GGC ATG G-3′, R 5′-GGC ATT AAA GCA GCG TAT CC-3′, tdTom WT/WT:-F 5′-AAG GGA GCT GCA GTG GAG TA-3′, R 5′-CCG AAA ATC TGT GGG AAG TC-3′.

### In vivo treatments

Tamoxifen (100 mg/kg; Sigma-Aldrich) was injected intraperitoneally (ip) for five consecutive days (P30 to P35) to induce recombination in vivo. The tamoxifen was dissolved in sunflower oil (S5007, Sigma-Aldrich).

### Analysis of transcriptomic resources

We queried available scRNA-seq data for *Mpc1* expression (*15*, *27*, *55*). For the expression of *Mpc1* in NSPCs, the original unsupervised clustering and pseudotime subgroups (S1 to S5) from Shin *et al.* were further divided in three subgroups: S1-S2, S3, and S4-S5, reflecting, respectively, a more quiescent to proliferative state.

### Virus preparation

CAG-Cre-GFP virus was produced as previously described (*67*). Briefly human embryonic kidney 293 T cells were transfected with a pCAG-GFPcre, pCMV-gp, and pCMV-vsvg using Lipofectamine 2000 (no. 10696153, Thermo Fisher Scientific) in Opti-MEM (no.11520386, Thermo Fisher Scientific). Forty-eight hours after transfection, the virus was collected by filtering the cell culture medium through a 0.22-μm filter. The filtrate was then concentrated twice using ultracentrifugation at 19,400 rpm. The viral pellet was resuspended in phosphate-buffered saline (PBS) and used for in vitro experiment and stereotaxic injections.

### Cell culture

Adult mouse NSPCs from hippocampus of 7-week-old C57/Bl6 (MPC1 WT) or *Mpc1* fl/fl (MPC1 cKO) male mice were isolated as previously described (*68*). For propagation, cells were cultured as spheres in Dulbecco’s modified Eagle medium (DMEM)/F12 GlutaMAX (no. 31331-028, Invitrogen) supplemented with N2 (no. 17502048, Invitrogen), human epidermal growth factor (EGF, 20 ng/ml; no. AF-100-15, Peprotech), human fibroblast growth factor (FGF)–basic (20 ng/ml; no. 100-18B, Peprotech), and heparin (5 μg/ml; no. H3149-50KU, Sigma-Aldrich). Medium contained as well an antibiotic-antimycotic (no. 15240062, Invitrogen). Medium was changed every second day. For proliferation experiments, cells were plated (42,000 cells/cm^2^) on glass coverslip (no. 10337423, Thermo Fisher Scientific) coated with poly-l-ornithine (50 μg/ml; no. P36655, Sigma-Aldrich) and laminin (5 μg/ml; no. L2020, Sigma-Aldrich) and grown as described above.

Quiescence was induced as previously described (*18*). Proliferating NSPCs (40,000 cells/cm^2^) were plated on coated glass coverslip or coated plastic cell culture well plate. Twenty-four hours later, medium was changed to quiescence medium based on DMEM/F12 GlutaMAX, supplemented with N2, human FGF-basic (20 ng/ml), heparin (5 μg/ml), and bone morphogenetic protein 4 (50 ng/ml; no. 5020BP, R&D Systems). Seventy-two hours after induction, cells were fully quiescent and used for subsequent experiments. For UK5099 experiment, quiescence was fully established for 3 days, and then cells were washed off, trypsinized, and plated (220,000 cells/cm^2^) on coated coverslips in fresh quiescence medium.

For comparison, proliferative NSPCs were plated at a lower density (13,000 cells/cm^2^) under proliferative conditions. The quiescence state was reversed by collecting quiescent NSPCs and replating them in proliferative conditions (50,000 cells/cm^2^) for 3 days (Fig. 2A). 5-ethynyl-2′-deoxyuridine (EdU) pulse was performed by incubating quiescent NSPCs with 10 μM EdU for 1 hour at 37°C before fixation.

For differentiation experiments, cells were plated on glass coverslip (no. 10337423, Thermo Fisher Scientific) or cell culture plates coated with poly-l-ornithine (50 μg/ml; no. P36655, Sigma-Aldrich) and laminin (5 μg/ml; no. L2020, Sigma-Aldrich) and cultured with a medium containing one-fifth of human EGF and human FGF-basic, i.e., 2 ng/ml. After 3 days, medium was change to medium without any human EGF and human FGF-basic. Cells were fixed for 15 to 20 min with 4% paraformaldehyde (PFA) after 6 to 7 days of differentiation. Cells were washed twice for 10 min with PBS and stored at 4°C before staining.

### Treatment with drugs and aspartate

Proliferative NSPCs were treated with either 1 or 10 μM UK5099 (no. PZ0160, Sigma-Aldrich) for 72 hours. For quiescence experiments, NSPCs were treated with 1 or 10 μM UK5099 (no. PZ0160, Sigma-Aldrich), 1 μM lactate dehydrogenase A inhibitor (GSK 2837808A, Tocris, 5189/10), and 1, 2.5, or 5 mM aspartate (no. 1690.2, Roth) simultaneously with quiescence induction. For exit experiments, quiescent NSPCs were treated with UK5099 after establishment of quiescence.

For differentiation experiments, proliferative NSPCs were treated with 1 or 10 μM UK5099 for 48 hours. Then, cells were washed off and plated (65,000 cells/cm^2^) on coated coverslips in media containing one-fifth of the growth factors and indicated UK5099 concentration. Similar to UK5099 treatment, 1, 2.5, or 5 mM aspartate (no. 1690.2, Roth) was used during the induction of quiescence.

### Virus infection in vitro

Proliferative NSPCs were plated (58,000 cells/cm^2^) on a cell culture plate coated with poly-l-ornithine (50 μg/ml; no. P36655, Sigma-Aldrich) and laminin (5 μg/ml; no. L2020, Sigma-Aldrich). One day after seeding, 0.5 μl of RV Cre-GFP virus was added to cells. Two days after virus infection, cells (40,000 cells/cm^2^) were replated on a glass coverslip in a proliferative medium. Twenty-four hours after, medium was changed to a quiescent medium (see the "Cell culture" section). After 3 days of quiescence, cells were fixed using 4% PFA.

### Extracellular lactate measurement

After 3 days of quiescence, medium was collected, spun down at 300*g* for 5 min, and snap-frozen before measurements. Lactate concentration was assessed using a buffer containing 0.33 M glycine-semicarbazide (Sigma-Aldrich, S2201), 0.015 M NAD (Roche, 12127973001), and lactate dehydrogenase (70 U/ml; Roche, 10127876001). Lactate concentration was assessed in an indirect way—by measuring the conversion of NAD to NADH measured at 340 nm.

### Metabolic fuel flux assays

OCR and ECAR were measured with the Seahorse XFe96 Analyzer (Agilent). Four thousand NSPCs per well were plated on 96-well Seahorse XF96 cell culture microplates with proliferative medium. After 24 hours, quiescence was induced (see the “Cell culture” section). After three full days of quiescence condition, cells were carefully washed with unbuffered DMEM (no. 103575-100, Agilent) supplemented with 10 mM glucose (no. 103577-100, Agilent), 2 mM glutamine (no. 103579-100, Agilent), and 1 mM pyruvate (no. 103578-100, Agilent), and a final volume of 180 μl of assay media was added to cells. The plate was allowed to equilibrate in a non-CO_2_ incubator at 37°C for 1 hour before analysis.

Mito Fuel Flex Test (no. 10360-100, Agilent) was performed according to manufacturer’s protocol. Baseline OCR was monitored for 14 min followed by different combinations of inhibitor injections as reported in Table 1 with OCR measurements. The Mito Fuel Flex Test inhibits the import of the three main metabolic substrates—pyruvate, glutamine, and fatty acids—using UK5099 (2 μM; no. PZ0160, Sigma-Aldrich), BPTES (3 μM; no. SML0601, Sigma-Aldrich), and etomoxir (4 μM; no. E1905, Sigma-Aldrich), respectively.

**Table 1.** Combination of inhibitors.

Metabolic test	Injection no. 1	Injection no. 2
Pyruvate dependency	UK5099	Etomoxir + BPTES
Pyruvate capacity	Etomoxir + BPTES	UK5099
Glutamine dependency	BPTES	UK5099 + etomoxir
Glutamine capacity	UK5099 + etomoxir	BPTES
Fatty acid dependency	Etomoxir	UK5099 + BPTES
Fatty acid capacity	UK5099 + BPTES	Etomoxir

Dependency was calculated as

$$\mathrm{Dependency}\;(\%)=\left(\frac{\text{Baseline OCR}-\text{target inhibitor OCR}}{\text{Baseline OCR}-\text{All inhibitors OCR}}\right)*100$$

Capacity was calculated as

$$\mathrm{Capacity}\;(\%)=\left[1-\left(\frac{\text{Baseline OCR}-\text{other 2 inhibitors OCR}}{\text{Baseline OCR}-\text{All inhibitors OCR}}\right)\right]*100$$

Flexibility was calculated as

Flexibility(%)=%Capacity−%dependency

To assess the effect of MPC1 inhibition, baseline OCR and ECAR were measured after 14 min in quiescent NSPCs. Different combinations of drugs—UK5099 (1 μM), etomoxir (20 μM), BPTES 3 (μM), etomoxir/BPTES, UK5099/etomoxir, or UK5099/BPTES—were injected, and OCR and ECAR were measured over 40 min. The change in OCR or ECAR were calculated as (ΔOCR) = (OCR following drug injection − OCR at baseline) and (ΔECAR) = (ECAR following drug injection − ECAR at baseline).

To normalize each well to cell numbers, after the run, cells were incubated with Hoechst (1:2000; no. H1399, Sigma-Aldrich) for 15 min and fixed with 4% PFA for 15 min. Cells were then washed with PBS, and all the wells were imaged with a Thunder microscope (Leica, DMi8). Hoechst signal was thresholded, and the area cover was measured using the software Fiji (ImageJ, 2.0.0).

### Electroporation and FACS separation

Proliferating MPC1 WT and MPC1 cKO NSPCs were tranfected with a plasmid encoding mCherry fused to Cre-recombinase (7 μg/μl, pCAGmCherryCre) using a 4D-Nucleofector X Unit EA (no. LZ-AAF-1002X, RUWAG) and the P3 primary cell 4D-Nucleofector X solution (no. LZ-V4XP-3024, RUWAG). NSPCs were cultured in normal proliferative medium for 48 hours before being subjected to FACS. Cells were trypsinized and resuspended in EDTA-Dulbecco’s PBS (no. E8008, Merck Millipore) and kept in ice until the sorting. mCherry-positive cells were sorted on a MoFlo Astrios EQ cell sorter (Beckman Coulter) and collected in proliferative medium or directly frozen on dry ice.

### Immunocytochemistry

To visualize MPC1 and mitochondria, cells plated on a glass coverslip were fixed with 4% PFA and 0.2% glutaraldehyde for 15 min. For all the other stainings, cells were fixed with 4% PFA for 15 to 20 min. Cells were washed twice for 10 min with PBS and stored at 4°C before staining. Cells were blocked for 45 min with blocking buffer containing 0.2% Triton X-100 (no. X100, Sigma-Aldrich) and 3% donkey serum (no. S30, Sigma-Aldrich) in tris-buffered saline [(TBS), 50 mM tris-Cl (pH 7.4), and 150 mM NaCl] and incubated with the indicated primary antibodies diluted in blocking buffer at 4°C overnight: mouse-pH3 (1:2000; Abcam, ab14955), rabbit-Ki67 (1:500; Abcam, ab15580), rabbit-MPC1 (1:100; Sigma-Aldrich, HPA045119), rabbit- TOMM20 (1:500; Abcam ab186735), and mouse-Map2b (1:500; Sigma-Aldrich, M2320). Cells were washed three times in TBS for 10 min and incubated with secondary antibodies in blocking buffer for at least 1 hour at room temperature, protected from light (Alexa Fluor; donkey anti-mouse 488, donkey anti-rabbit 488, and donkey anti-rabbit 647, 1:250). Cells were washed twice for 10 min with TBS. Nuclei were stained with 4′,6-diamidino-2-phenylindole (DAPI, 1:5000; no. D9542, Sigma-Aldrich) for 5 min and washed twice with TBS. Coverslips were mounted with a self-made polyvinyl alcohol–DABCO–based mounting medium. To detect EdU, the manufacturer’s protocol was followed (Click-iT Plus EdU Alexa Fluor 647 imaging kit, no. 15224959, Invitrogen).

### Image acquisition and analyses

All images used to assess the ablation of MPC1, the quantification of MAP2AB, and the mitochondrial shape in MPC1 WT and MPC1 cKO were acquired with a confocal microscope (Zeiss, LSM780) with a 40× or 63× objective. Images were analyzed using the software Fiji (ImageJ 2.0.0). Dentritic length was measured using the plugin “Simple Neurite Tracker” on Fiji. All images to assess the proliferation were acquired in a blinded manner with an epifluorescent microscope (Nikon 90i or Leica DMi8) with a 20× or 40× objective. Ten pictures per coverslip were taken and analyzed using Fiji (ImageJ 2.0.0) in a blinded manner. For DAPI area cover, 56 tiles with a 10× objective were acquired with an epifluorescent microscope (Leica DMi8). Area over was measured in thresholded images. For mitochondrial analysis, serial z-stacks (0.3 μm) were taken using a confocal microscope (Zeiss, LSM780) with a 63× objective. Mitochondrial length was measured using the freehand tool in Fiji, as previously described (*21*).

### RNA-seq and qRT-PCR on cultured NSPCs

For RNA-seq, MPC1 WT and MPC1 cKO NSPCs were tranfected with a plasmid pCAGmCherryCre and sorted according to mCherry (as described above). Directly after the sort cells were collected and frozen down before RNA extraction. RNA was extracted using an RNeasy micro kit (Qiagen) according to the manufacturer’s protocol. RNA-seq data were generated and analyzed by Alithea Genomics SA (Switzerland).

For in vitro samples, NSPCs were collected and snap-frozen on dry ice. RNA was extracted using RNeasy mini kit (no. 74134, Qiagen) according to the manufacturer’s protocol, followed by complementary DNA synthesis using a SuperScript IV system (no. 15327696, Invitrogen). qRT-PCR was performed using PowerSYBR Grenn PCR Master Mix (Thermo Fisher Scientific, no. 10658255). The following primers were used:

*Mpc1*: 5′-GAC TAT GTC CGG AGC AAG GA-3′ (forward) and 5′-TAG CAA CAG AGG GCG AAA GT-3′ (reverse); *Mpc2*: 5′-TGC TGC CAA AGA AAT TG-3′ (forward) and 5′-AGT GGA CTG AGC TGT GCT GA-3′ (reverse); *Got1*: 5′- CTT TAA GGA GTG GAA AGG TAA C-3′ (forward) and 5′- GAG ATA GAT GCT TCT CGT TG-3′ (reverse); *Got2*: 5′- TAT CAA AAA TCC CAG AGC AG-3′ (forward) and 5′-ATT CTT TTT CTT CAC CAC GG -3′ (reverse); *Asns*: 5′- CCA AGT TCA GTA TCC TCT CC -3′ (forward) and 5′- TAA TTT GCC ACC TTT CTA GC-3′ (reverse); *Slc1a3*: 5′- TCA TCT CCA GTC TCG TCA CA-3′ (forward) and 5′- CAC CAC AGC AAT GAT GGT AGT A-3′ (reverse); *Ppat*: 5′-CCA GAT AGT ATG TTT GAA GAC C-3′ (forward) and 5′-CAC TTT GTT GCA TAT CCC AG -3′ (reverse); *Pfas*: 5′-TTG AAT GCA CTG GAA ATC TG-3′ (forward) and 5′-CGA TCA TCC ACT AGT ACA ATT C-3′ (reverse); *Paics*: 5′-GAA TAG CAA CTG GGT CTT TC-3′ (forward) and 5′-ACT TCA GTC TGC CCT ATA AC-3′ (reverse); *Adsl*: 5′-GAC AAG ATG GTG ACA GAA AAG-3′ (forward) and 5′-AAT CTG CTG TTT CTC AAA GG-3′ (reverse); *Atic*: 5′-GCA TGG TCT ATG ACC TCT AC-3′ (forward) and 5′-AAT ACC ATC CGA CAC TTC TC-3′ (reverse); *Cad*: 5′-CTT CTT CAG TGT CCA GTT TC-3′ (forward) and 5′-CTA GAA ATA CAT CGA AGA GCA G-3′ (reverse); *Umps*: 5′-AAA AGC AGT ATG AAA GTG GC-3′ (forward) and 5′-CTG TAA GCC TTT CAC AAC TC-3′ (reverse); and β*-actin*: 5′-GCC CTG AGG CTC TTT TCC AG-3′ (forward) and 5′-TGC CAC AGG ATT CCA TAC CC-3′ (reverse). β*-actin* was used as a housekeeping gene to normalize the obtained cycle threshold (CT) values, and each sample gene expression was calculated using the comparative ΔΔCT method.

### qRT-PCR on brain tissue

Total RNA from hippocampal tissue was isolated using TRIzol reagent (Thermo Fisher Scientific) method, and RNA concentration was determined using NanoDrop One (Thermo Fisher Scientific). Reverse transcription was performed with 500 to 1000 ng of deoxyribonuclease-treated (Promega, RQ1 M610A) total RNA using GoScript reverse transcriptase (Promega A6001). The qRT-PCR was done on a Bio-Rad CFX Connect optics module. CT values were normalized to β-actin.

*Mpc1*: 5′-GAC TAT GTC CGG AGC AAG GA-3′ (forward) and 5′-TAG CAA CAG AGG GCG AAA GT-3′ (reverse); *Mpc2*: 5′-TGC TGC CAA AGA AAT TG-3′ (forward) and 5′-AGT GGA CTG AGC TGT GCT GA-3′ (reverse); β*-actin*: 5′-GGCTGTATTCCCCTCCATCG-3′ (forward) and 5′-CCAGTTGGTAACAATGCCATGT-3′ (reverse).

### Protein extraction and Western blotting

Hippocampal brain and cellular extracts were homogenized in radioimmunoprecipitation assay lysis buffer [50 mM tris-HCl (pH 7.5), 150 mM NaCl, 1% NP-40, 0.5% sodium deoxycholate, 0.1 SDS, 1 mM EDTA, 10% glycerol, and protease inhibitors] and resolved by SDS–polyacrylamide gel electrophoresis in 8 to 15% polyacrylamide gels. The concentration of the proteins was determined by Bradford protein assay using bovine serum albumin as standard and analyzed by Western blotting with specific antibodies.

Antibodies used were as follows: rabbit-MPC1 (Sigma-Aldrich, HPA045119), mouse-MPC2 (Merck, MABS1914-25UG), goat-voltage dependent anion channel protein (VDAC) (Santa Cruz Biotechnology, sc-8829), mouse-HSP70 (Invitrogen, MA3-028), total OXPHOS antibody cocktail (Abcam, MS604-300), anti–immunoglobulin G (IgG)–rabbit–horseradish peroxidase (HRP) (Dako, P0217), anti–IgG-mouse-HRP (Dako, P0447), and anti–IgG-Goat-HRP (Santa Cruz Biotechnology, sc-2304).

### Tissue preparation and immunohistochemistry

All experimental mice were deeply anesthetized with sodium pentobarbitone (6 mg/100 g body weight, ip) at different time points after tamoxifen injection (P33, P38, P60, and 10 to 12 months) and immediately perfused intracardiacally with fresh 4% PFA in 0.1 M PBS (pH 7.4). Brains were postfixed overnight at 4°C. Coronal sections of 50 and 70 μm were cut using a vibratome (Leica) and stored at 4°C in 1× PBS supplemented with 0.02% sodium azide. For immunohistochemical analysis, sections were permeabilized for 1 hour in PBS containing 0.3% Triton X-100 and 10% donkey or goat serum and then immunolabeled overnight at 4°C on an orbital shaker, using the following primary antibodies: mouse-NeuN (1:200; Merck-Millipore, MAB377), chicken-GFP (1:500; Abcam, ab13970), rabbit-DCX (1:400; Cell Signaling Technology, 4604), guinea pig-DCX (1:100; Merk-Millipore, AB2253), and rabbit-Ki67 (1:400; Abcam, ab16667). For rabbit-MPC1 (1:200; Sigma-Aldrich, HPA045119) and rat-Ki67 (1:400; Thermo Fisher Scientific, 14-5698-82) staining, the antibodies were incubated for 72 hours at 4°C. After the primary antibody incubation, the sections were washed again three times in PBS for 5 min and incubated for 2 hours at room temperature with fluorescent secondary antibodies (Alexa Fluor, goat anti-mouse 647; donkey anti-mouse 647, goat anti-rabbit 488, donkey anti-rabbit 488, and donkey anti-guinea pig 647; donkey anti-goat 488, donkey anti-chicken 488, and donkey anti-rat 647, 1:300) diluted in PBS. Last, nuclei were counterstained with DAPI (1:10,000; Invitrogen) and then washed before mounting with a FluorSave reagent (Merck-Millipore).

### Metabolomics analysis

For untargeted metabolic profiling, adherent cells were washed with a solution containing 75 mM ammonium carbonate (no. 379999-10G, Sigma-Aldrich), and the pH was buffered at 7.4 using acetic acid. After removing the liquid, cells were frozen by holding the place on dry ice. Extraction of metabolites was performed by adding ice-cold acetonitrile (no. 34967, Sigma-Aldrich), methanol (no. 34966, Sigma-Aldrich), and water (no. W6-500, Thermo Fisher Scientific) (4:4:2, v/v) solvent mixture to cells. The extracts were centrifuged at 13,000 rpm for 2 min before analysis.

Nontargeted metabolomics analysis was performed by flow-injection–time-of-flight mass spectrometry (MS) on an Agilent 6550 QTOF system (PMID21830798). The instrument was set to scan in full MS at 1.4 Hz in negative ionization, 4-GHz high-resolution mode, from 50 to 1000 mass/charge ratio. The solvent was 60:40 isopropanol/water supplemented with 1 mM NH_4_F at pH 9.0, as well as 10 nM hexakis (1H, 1H, 3H-tetrafluoropropoxy) phosphazine and 80 nM taurochloric acid for online mass calibration. The injection sequence was randomized. Data were acquired in profile mode, centroided, and analyzed with Matlab (The Mathworks, Natick). Missing values were filled by recursion in the raw data. Upon identification of consensus centroids across all samples, ions were putatively annotated by accurate mass and isotopic patterns. Starting from the HMDB v4.0 database, we generated a list of expected ions, including deprotonated, fluorinated, and all major adducts found under these conditions. All formulas matching the measured mass within a mass tolerance of 0.001 Da were enumerated. As this method does not use chromatographic separation or in-depth MS2 characterization, it is not possible to distinguish between compounds with identical molecular formula. Principal components analysis and statistical comparison between the groups were performed using RStudio. Pathway enrichment analysis was performed using MetaboAnalyst 5.0.

### Virus injections in vivo

One-month-old (P30) recombined MPC1cKO-tdTom and control MPC1 WT-tdTom mice were anesthetized using isoflurane at 5% (w/v), placed in a small animal stereotaxic frame (David Kopf Instruments), and maintained at 2.5% isoflurane (w/c) for the duration of surgery. Corneal and pinch reflexes were regularly tested to confirm anesthetic depth. Lacryvisc (Aicon, Switzerland) was applied to prevent corneal drying and lidocaine applied topically to the skin overlying the skull. After exposing the skull under aseptic conditions, a small hole was drilled in the skull overlying the DG. RV Cre-GFP was injected monolaterally into the DG from the bregma (anterior posterior, −2 mm; lateral +1.5 mm, 2.3 dorsal ventral mm from skull) at a rate of 100 nl/min^−1^ (1.5-μl total volume) using a Hamilton syringe and a CMA400 pump (CMA System, Kista, Sweden). Mice were euthanized 4 weeks after virus injections.

### Confocal microscopy acquisition and image analysis

All images were collected on a Leica confocal imaging system (TCS SP8) with a 20× or 63× oil immersion objective, Leica Thunder imaging system (DMi8) with a 20× objective, and Spinning Disk confocal imaging system (Nikon Ti2/Yokogawa CSU-W1) with 60**×** and 100× oil immersion objectives. For the quantification of recombined tdTom-positive cells with specific markers, serial sections starting from the beginning of the DG were used. Quantification of tdTom-positive and Ki67-positive cell labeling with different markers, DCX and NeuN, was performed using the software Fiji (ImageJ 2.0.0). To confirm Ki67, DCX, and NeuN colocalization with tdTom, single optical sections at 63× magnification were used.

For MPC1 quantification in tissue, z-stacks of single Nestin-GFP cells positive or negative for Ki67 (63× objective) were acquired. Quantification of MPC1 and GFP signal was performed using IMARIS software (Bitplane 9.9.1). First, the GFP volume reconstruction was performed using surface plugin. To confirm the signal of MPC1 in GFP-expressing cells, MPC1 signal was masked in GFP-positive cells using mask plugin. Last, MPC1 volume reconstruction of masked signal was performed using surface plugin. Eighteen to 26 GFP-positive cells were analyzed per group.

For Sholl analysis, the Cre virus–transfected GFP and tdTom double-positive newborn neurons were imaged with a 63× [0.75 numerical aperture (NA)] objective. Z-stacks were taken at 1-μm intervals, and dendrites were traced using the Neurolucida software (version 10, mbs Bioscience). Fifteen to 18 neurons from four mice per group were analyzed. Dendritic spine density and spine morphology were assessed as previously described (*69*, *70*) with little adjustments. Briefly, dendrites of 20 to 30 Cre virus–transfected GFP and tdTom double-positive newborn neurons were imaged using a 63× (2.5 NA) objective. The dendritic length and the number of spines were analyzed using the software Fiji (ImageJ 2.0.0). Spine density was expressed as the number of the spines divided by dendritic length. Spine morphology was classified in two groups on the basis of the maximal diameter of the spine head, as measured on maximal projections with Fiji (ImageJ 2.0.0). Immature spines (thin spines) were defined as 0.25 to 0.6 μm and mature spines (mushroom) >0.6 μm. The percentage of each type of dendritic spine was then expressed by neuron for each mouse (20 to 30 neurons per mouse, four mice per group). The data were expressed as the ratio between immature spines and mature spines. All images were analyzed in a blinded manner.

### Statistical analyses

Normality of data was tested using the Shapiro-Wilk test. When samples followed normal distribution, unpaired Student’s *t* test was conducted for comparison of two samples, else nonparametric Mann-Whitney test was used (Fig. 2E and fig.S2D). For metabolomics data, *P* value was adjusted for multiple comparisons. For comparisons with more than two groups, one-way analysis of variance (ANOVA) was performed followed by Holm-Sidak’s multiple comparison tests. When samples did not follow normality test, Kruskal-Wallis test was computed. For dendritic complexity in Sholl analysis, area under the curve was calculated followed by unpaired Student’s *t* test. All analyses were performed using GraphPad Prism 8.0.2 software.

### Illustration software

For illustration schemes, Biorender software (Biorender, 2021) and Adobe Illustrator (version 25.0, Adobe Inc.) were used.

## Notes

View/request a protocol for this paper from *Bio-protocol*.
