# Supplementary material for: Mitochondrial pyruvate metabolism regulates the activation of quiescent adult neural stem cells
Source: Sci Adv. 2023 Mar 1;9(9):eadd5220. doi: 10.1126/sciadv.add5220 (PMC9977184; doi:10.1126/sciadv.add5220)
Supplement: Supplementary file 1 — Figs. S1 to S6 [file sciadv.add5220_sm.pdf]

Science Advances
AAAS

Supplementary Materials for

Mitochondrial pyruvate metabolism regulates the activation of quiescent adult neural stem cells

Francesco Petrelli *et al.*

Corresponding author: Jean-Claude Martinou, jean-claude.martinou@unige.ch;
Marlen Knobloch, marlen.knobloch@unil.ch

Sci. Adv. **9**, eadd5220 (2023)
DOI: 10.1126/sciadv.add5220

This PDF file includes:

Figs. S1 to S6

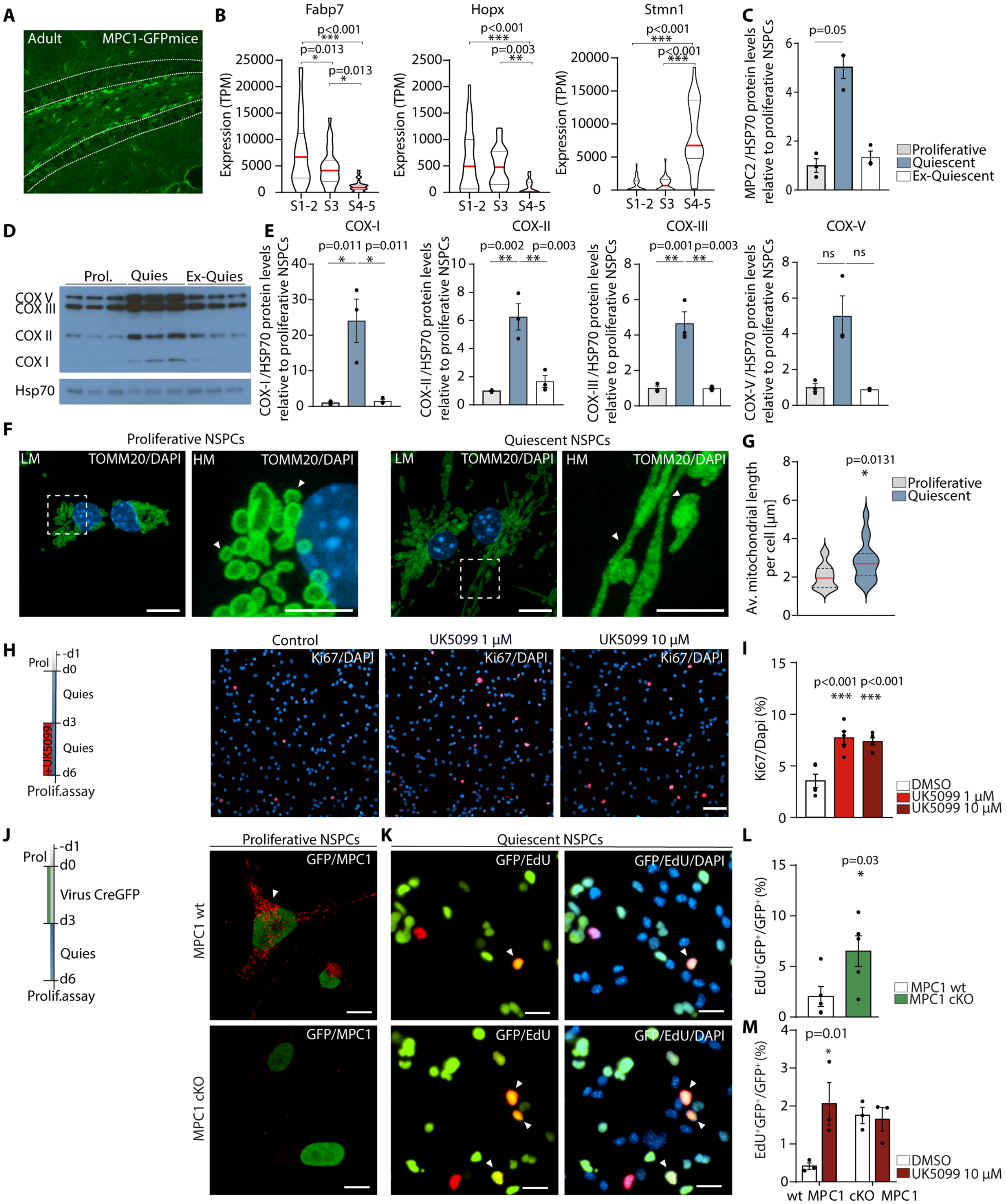

Fig. S1: MPC is dynamically regulated with activity state and its transport function is required for NSPC quiescence, related to figure 1.

(A) Representative sagittal confocal image (40X) of adult DG of MPC1-GFP mice (Gensat data, http://www.gensat.org/imagenavigator.jsp?imageID=26000). Dotted line outlines the granular zone of the DG. **(B)** Violin plots of Fabp7, Hopx and Stmn1 expression in quiescent (S1-2), activated NSPCs (S3) and intermediate progenitor cells (S4-5). Data queried from Shin et al. (*15*). Red line represents mean. *p<0.05, **p<0.01, ***p< 0.001

(one-way ANOVA followed by post-hoc test). **(C)** Quantification of MPC2 protein in proliferative (grey), quiescent (light blue) and ex-quiescent (grey) NSPCs. MPC2 expression was normalized to HSP70 levels and expressed as fold change to proliferative NSPCs. Bars represent mean ± SEM., n=3 biological replicates (Kruskal wallis test followed by post-hoc test). **(D)** Representative western blots of the different complexes of the oxidative phosphorylation chain: cytochrome c oxydase (COX)-I (18 kDa), COX-II (30 kDa), COX-III (48 kDa), COX-V (55 kDa) and HSP70 (70 kDa). **(E)** Quantification of COX-I, COX-II, COX-III and COX-V in proliferative (grey), quiescent (light blue) and ex-quiescent (grey) NSPCs. Protein levels were normalized to HSP70 levels and expressed as fold change to proliferative NSPCs. Bars represent mean ± SEM.*p< 0.05 ,**p<0.01, n=3 biological replicates (one-way ANOVA followed by post-hoc test, for COX-V, non-parametric Kruskal wallis test was used.). **(F)** Representative LM and HM confocal images of TOMM20 (green) and DAPI (blue) immunostaining in proliferative and quiescent NSPCs. Arrowhead show mitochondria shape. Scale bars: 10 µm for LM and 5 µm for HM. **(G)** Quantification of mitochondrial length. *p<0.05. n=15 cells from 2 CS. (Unpaired Student t-test). **(H)** Left: Experimental outline of UK5099 treatment after quiescence establishment. Right: Representative images of Ki67 (red) and DAPI (blue) immunostaining in quiescent NSPCs treated with UK5099. Scale bar: 50 µm. **(I)** Quantification of Ki67+ in DMSO (white) or UK5099 (light and dark red) treated quiescent NSPCs. Data represent mean ± SEM. ***p<0.001. n=5 CS from 2 independent experiments (one-way ANOVA followed by post-hoc test). **(J)** Left. Experimental outline of MPC1 knock out using a Cre-GFP virus. Right: Representative confocal images of GFP (green) and MPC1 (red) in MPC1-wt and MPC1-cKO proliferative NSPCs 2 days after Cre-GFP virus infection. (**K**) Representative images of GFP (green), EdU (red) in MPC1-wt and MPC1 cKO quiescent NSPCs. (**L**) Quantification of EdU+ GFP+ cells over GFP+ cells in MPC1 wt (white) and MPC1 cKO quiescent (green) NSPCs. Data represent mean ± SEM. *p<0.05. n=5 independent infections (Unpaired Student t-test). (**M**) Quantification of EdU+ GFP+ cells over GFP+ cells in wt MPC1 and cKO MPC1 quiescent NSPCs treated with UK5099 10 µM. Data represent mean ± SEM. *p<0.05. n=3 CS from one experiment (two-way ANOVA followed by post-hoc test).

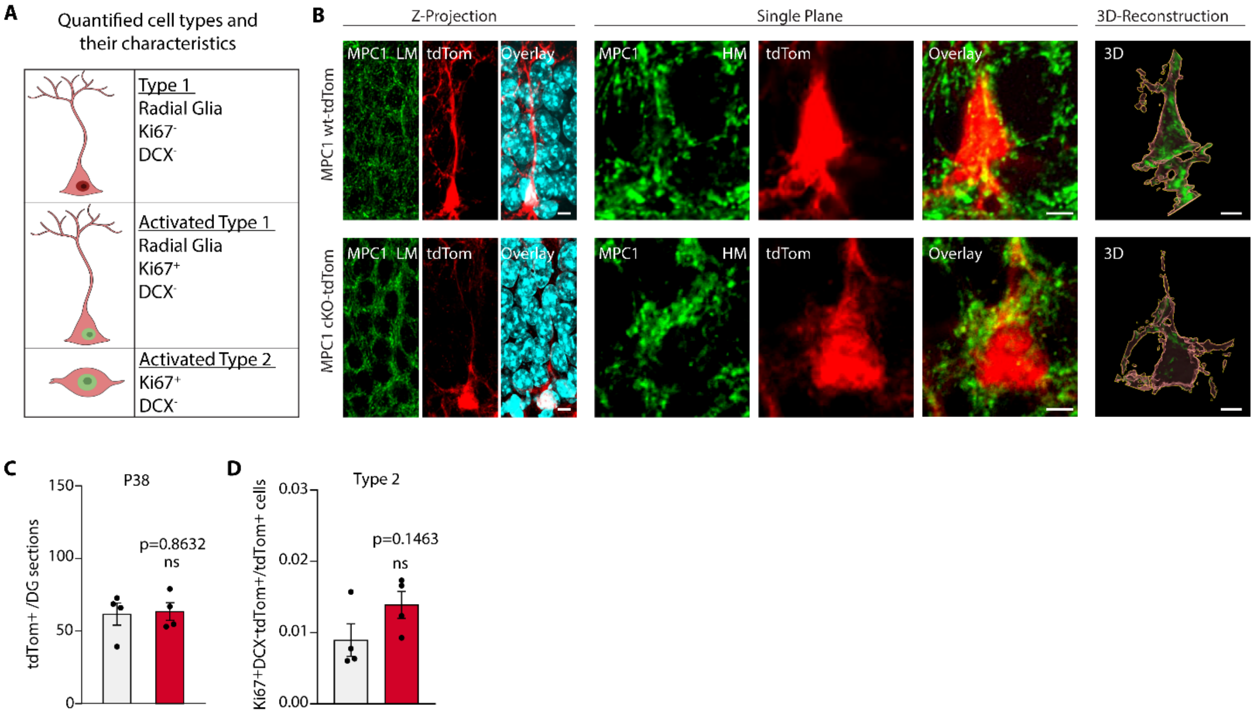

Fig. S2: Mpc1 deletion in NSPCs in vivo leads to increased numbers of progeny and triggers NSPC proliferation, related to figure 2.

(A) Scheme of NSPC types and their characteristics. (**B**) Representative low magnification (LM) and high magnification (HM) confocal images of MPC1 (green), tdTom (red) and DAPI (blue) in MPC1 wt-tdTom and MPC1 cKO-tdTom mice at P38. Right: 3D reconstruction of MPC1 staining within the tdTom signal. Scale bars: 5µm. (**C**) Quantification of the recombined tdTom+ cells in the DG of control MPC1 wt-tdTom (grey) and MPC1 cKO-tdTom (red) mice at P38. The graph shows the average number of tdTom+ recombined cells per DG sections (n=10 sections per mouse). Data show mean ± SEM. Not significant (ns), n=4 mice per group (unpaired Student's t-test). (D) Quantification of type-2 Ki67+ DCX- tdTom+ cells out of the total number of tdTom+ cells in the DG of control MPC1 wt-tdTom (grey) and MPC1 cKO-tdTom (red) mice. Data represent mean ± SEM. Not significant (ns), n=4 mice per group (unpaired Mann-Whitney t-test).

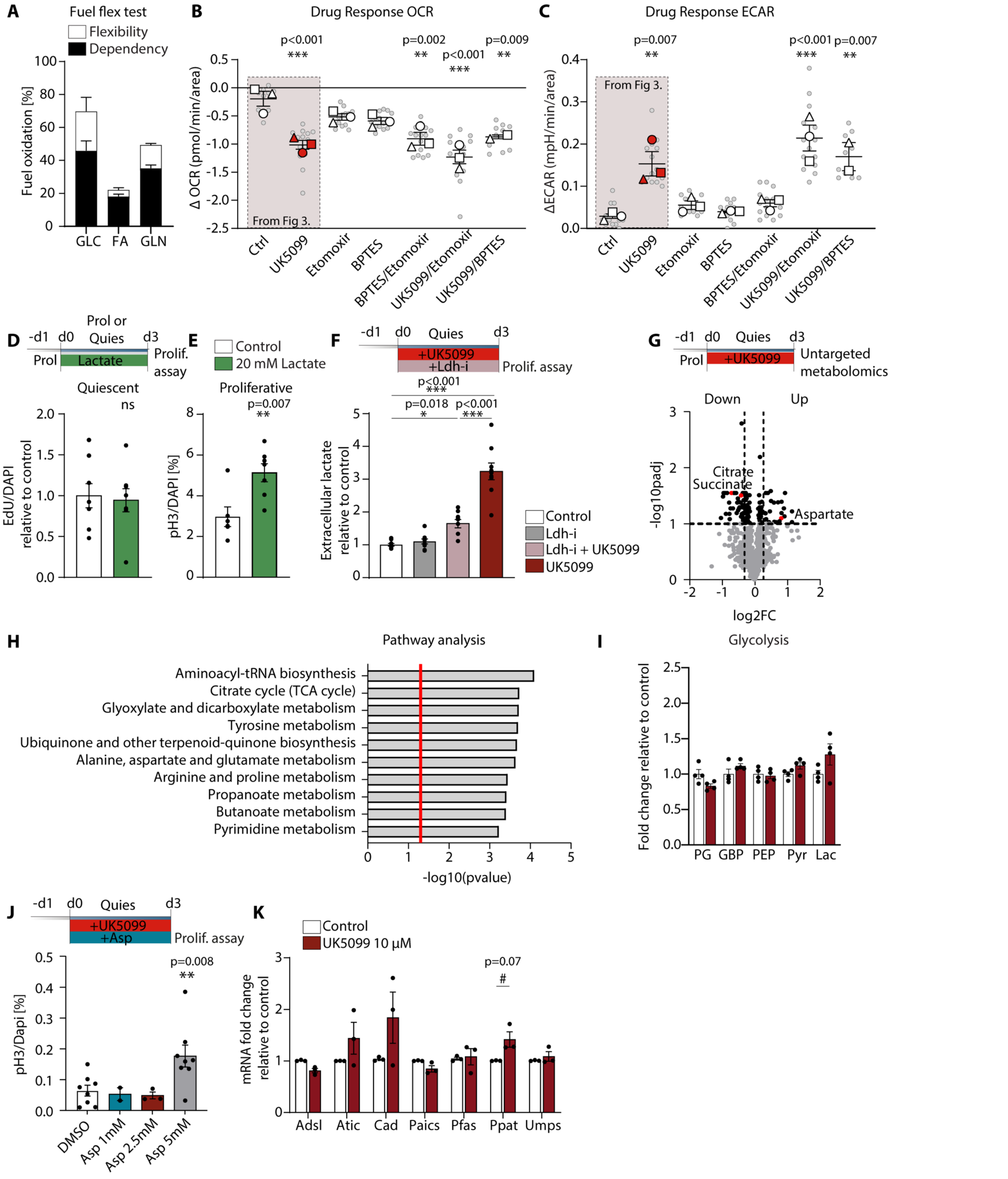

Fig. S3: MPC1 inhibition in quiescent NSPCs leads to increased intracellular aspartate levels, related to figure 3.

(**A**) Percent dependency (black) and flexibility (white) to oxidize glucose (GLC), glutamine (GLN) and fatty acids (FA) in quiescent NSPCs. Data represent mean ± SEM. n=3 independent experiments. (**B**) Difference in OCR after a single injection of medium (white), UK5099 1 µM (red), Etomoxir 4 µM, BPTES 3 µM, Etomoxir/BPTES, UK5099/BPTES or UK5099/Etomoxir in quiescent NSPCs. Data represent mean ± SEM. **p<0.01, ***p <0.001 n=3

biological replicates, gray dots represent individual wells of seahorse (one-way ANOVA with post-hoc test, compared to control). (**C**) Difference in ECAR after a single injection of medium (white), UK5099 1 μM (red), Etomoxir 4 μM, BPTES 3 μM, Etomoxir/BPTES, UK5099/BPTES or UK5099/Etomoxir in quiescent NSPCs. Data represent mean ± SEM, **p<0.01, ***p <0.001. n=3 biological replicates, gray dots represent individual well of seahorse (one-way ANOVA with post-hoc test, compared to control).(**D**) Top: Experimental outline of lactate treatment either during quiescence induction or in proliferative conditions . Bottom: Quantification of EdU positive cells in control (white) and 20 mM lactate-treated (green) quiescent NSPCs. Data represent mean ± SEM normalized to control condition. Not significant (ns), n=8 CS from 3 independent experiments. (Unpaired Student t-test).(**E**) Quantification of pH3+ cells in control (white) and 20mM lactate-treated (green) proliferative NSPCs. Data represent mean ± SEM.**p<0.01, n=6-7 CS from 3 independent experiments (Unpaired Student t-test). (**F**) Extracellular lactate concentration after 3 days of quiescence in control, Ldh-i, Ldh-i+UK5099 and UK5099 treated NSPCs. Data represent mean ± SEM normalized to control. *p<0.05,***p <0.001 n=9 samples from 3 independent experiments (One-way ANOVA followed by post-hoc test). (**G**) Representative Volcano plot of metabolomics data (fold change threshold = 1.2 and p-value adjusted=0.1) in control and UK5099 treated quiescent NSPCs. (**H**) Pathway enrichment analysis of significantly changed metabolites between control and UK5099-treated quiescent NSPCs. Red line: p-value=0.05. (**I**) Relative intensity of selected glycolytic metabolites in control and UK5099 treated quiescent NPSCs. Bars represent mean ± SEM. * adjusted p-value <0.1, N=4 biological replicates. PG: phosphoglycerate, GBP: Glyceric acid 1,3-biphosphate, PEP: phosphoenolpyruvate, Pyr: Pyruvate, Lac: Lactate. (**J**) Quantification of pH3 in quiescent NSPCs treated with several doses of aspartate. Data represent mean ± SEM. * p <0.01, n=2-8 CS from 3 independent experiments. (one-way ANOVA with post-hoc test compared to control.). (**K**) Relative mRNA expression of Adsl, Atic, Cad, Paics, Pfas, Ppat and Umps in control (white) and UK5099-treated (red) NSPCs. Genes are expressed as fold change to control NSPCs, bars represent mean ± SEM. # p=0.07, n=3 independent experiments. Statistics were computed on dCT values (unpaired Student t-test).

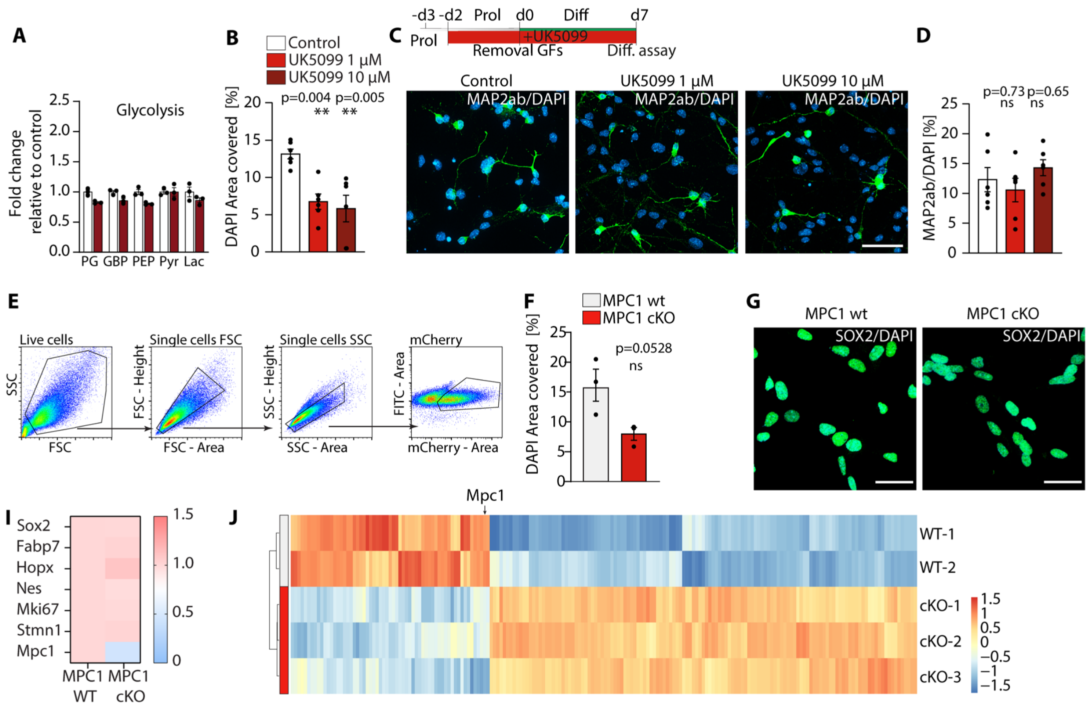

Fig. S4: MPC-deficient NSPCs can generate neurons through a shift in their metabolism, related to figure 4. (A) Relative intensity of selected glycolytic metabolites in control and UK5099 treated proliferative NSPCs. Bars represent mean ± SEM. * adjusted p-value <0.1, N=4 biological replicates. PG: phosphoglycerate, GBP: Glyceric acid 1,3-biphosphate, PEP: phosphoenolpyruvate, Pyr: Pyruvate, Lac: Lactate. (B) Quantification of DAPI area covered in control (white) and UK5099-treated (red) differentiated NSPCs. **p< 0.01. n=6 CS from 2 independent experiments. (one-way ANOVA followed by post-hoc test). (C) Top: experimental outline of differentiation in presence of UK5099. Bottom: Representative confocal images of neurons (MAP2ab, green) and DAPI (blue) in UK5099-treated differentiated cells. Scale bar: 20 µm. (D) Quantification of MAP2ab positive cells in control (grey) and UK5099-treated (red) cells. Not significant, n=6 CS from 2 independent experiments. (one-way ANOVA followed by post-hoc test). (E) Sorting and gating strategy of MPC1 wt and MPC1 cKO mcherry positive cells. (F) Quantification of DAPI area covered in MPC1 wt and MPC1 cKO cells. n=3 electroporations, mean ± SEM, unpaired Student's t-test. (G) Representative confocal images of SOX2 (green) and DAPI (blue) immunostaining in MPC1 wt and MPC1 cKO NSPCs. Scale bar: 50 µm. (H) Quantification of SOX2+ in MPC1 wt and MPC1 cKO NSPCs . Data represent mean ± SEM. Not significant (ns), n=3 electroporations (unpaired Student's t-test). (I) Heatmap of the fold change of the selected stemness markers and Mpc1 in MPC1 wt and MPC1 cKO NSPCs. (J) Heatmap showing the differentially expressed genes in MPC1-wt and MPC1-cKO NSPCs. Arrow highlights Mpc1 expression.

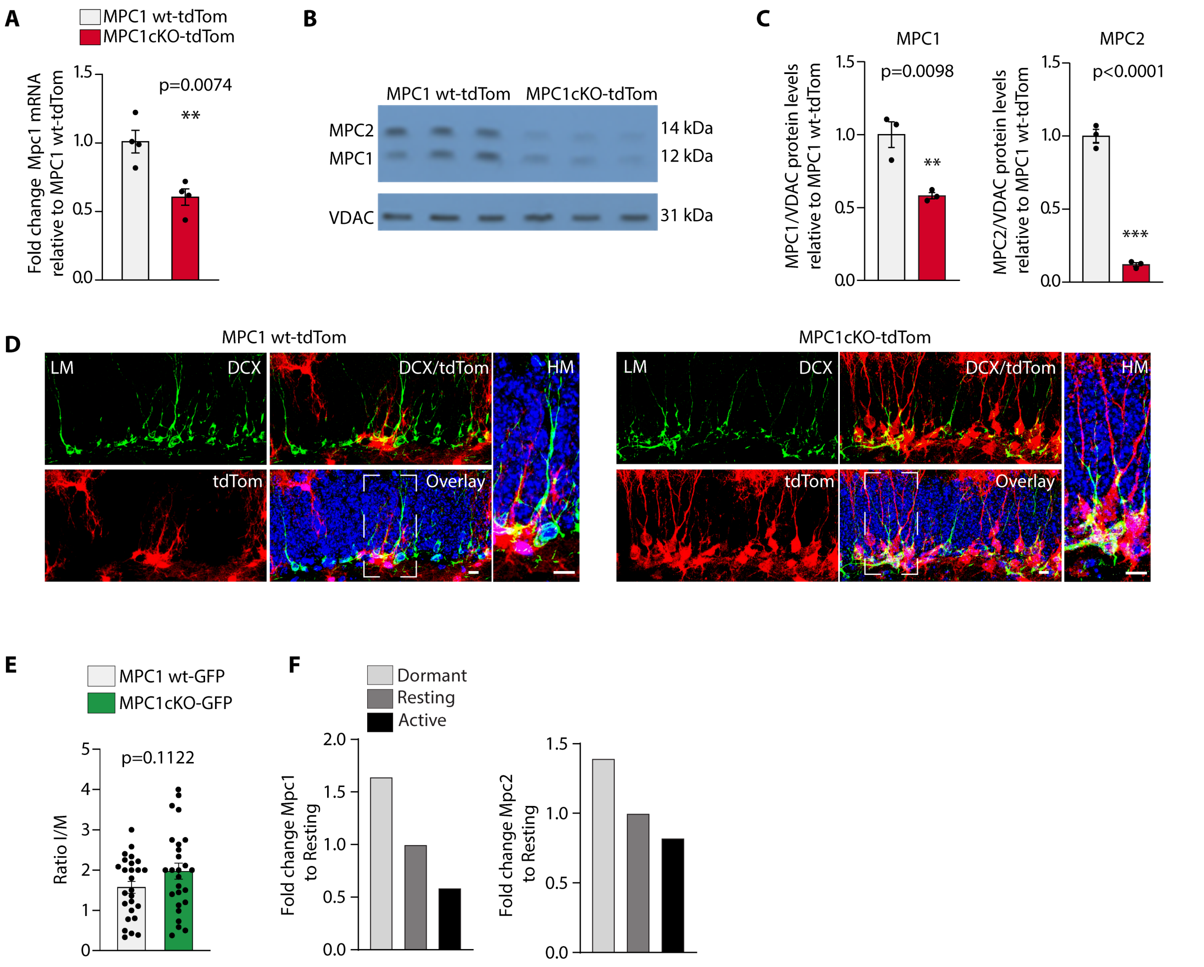

Fig. S5: Mpc1 deletion in NSPCs increases neurogenesis in vivo, related to figure 5.
(**A**) Relative mRNA expression levels of Mpc1 from hippocampus of control MPC1 wt-tdTom (grey) and MPC1 cKO-tdTom (red) mice. Data are expressed as fold changed compared to MPC1 wt-tdTom and represented as means ± SEM. **p<0.01, n=4 mice per group (unpaired Student's t-test). (**B**) Representative western blots of MPC1 (12 kDa), MPC2 (14 kDa) and protein voltage-dependent anion channel (VDAC, 31 kDa) in the hippocampus of control MPC1 wt-tdTom (n=3 mice) and MPC1 cKO-tdTom mice (n=3 mice). (**C**) Quantification of MPC1 and MPC2 protein expression in the hippocampus of control MPC1 wt-tdTom (grey) and MPC1 cKO-tdTom mice. MPC1 and MPC2 expression was normalized to HSP70 levels and expressed as fold change to MPC1 wt-tdTom mice. Data are shown as mean value ± SEM. **p<0.01, ***p<0.001, n=3 mice per group (unpaired Student's t-test). (**D**) Representative LM and HM confocal images of TAM-induced td-Tomato recombination (red), Doublecortin (DCX, green) and DAPI (blue) immunostaining at P60 in the DG of control MPC1 wt-tdTom (grey) and MPC1 cKO-tdTom (red) mice. Scale bars: 10 µm. (**E**) Ratio of the number of Immature/Mature (I/M) spines on Cre-induced tdTom+ newborn neurons in the DG of MPC1 wt-GFP and MPC1 cKO-GFP mice. Not significant (ns), n=4 mice per group, (unpaired Student's t-test). (**F**) Fold changes of Mpc1 and Mpc2 in dormant, resting and proliferating NSPCs. Processed scRNAseq data are from Harris et al. (*55*).

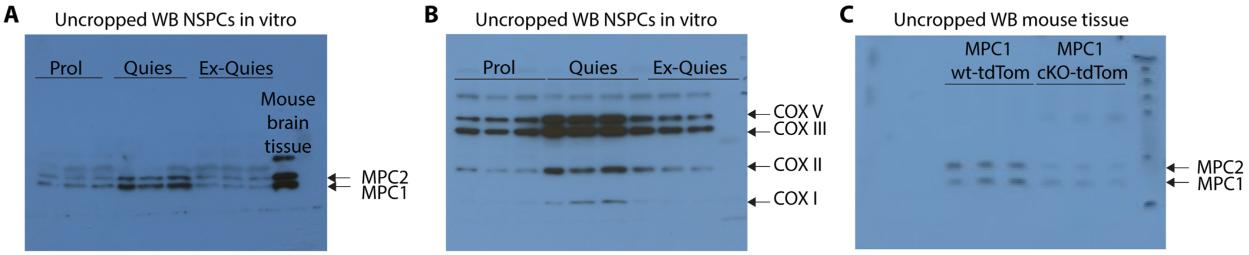

Fig. S6, Full size original Western blots, related to Fig.1, Suppl Fig.1 and Fig.5.
(**A**) Original blot of MPC1 (12 kDa) and MPC2 (14 kDa) in proliferative, quiescent and ex-quiescent NSPCs. Mouse brain tissue was loaded as a control. (**B**) Original blot of the different complexed of the oxidative phosphorylation chain: cytochrome c oxydase (COX)-I (18 kDa), COX-II (30 kDa), COX-III (48 kDa), COX-V (55 kDa). (**C**) Original blot of MPC1 (12 kDa) and MPC2 (14 kDa) in MPC1wt-tdTom and MPC1 cKO-tdTom brain tissues.
